# CytokineProfile: An Integrated Web Tool for Cytokine Profiling Analysis

**DOI:** 10.34133/csbj.0079

**Published:** 2026-05-07

**Authors:** Shubh Saraswat, Bira Arumndari Nurrahma, Philip A. Kern, Barbara S. Nikolajczyk, Xiaohua D. Zhang

**Affiliations:** ^1^ Department of Biostatistics, University of Kentucky, Lexington, KY 40536, USA.; ^2^ Department of Pharmacology and Nutritional Sciences, University of Kentucky, Lexington, KY 40536, USA.; ^3^ Department of Internal Medicine, University of Kentucky, Lexington, KY 40536, USA.

## Abstract

**Background:** Multiplex cytokine assays are widely used for biomarker discovery and disease profiling, but most existing web tools are optimized for metabolomics and require substantial data reformatting. A reproducible, end-to-end computational platform tailored to cytokine analysis is needed. **Methods:** We developed CytokineProfile, a web-based analytic tool that accepts standard tabular formats and guides users through data preview, quality control, missing-value handling, and filtering. Analytical modules include exploratory visualization (boxplots, correlation matrices, volcano plots, and dual-flashlight plots combining log fold change with strictly standardized mean difference), univariate tests (analysis of variance and normality-informed 2-sample tests), multivariate modeling (principal component analysis, sparse partial least squares discriminant analysis, and multilevel/multivariate integration variants), and machine-learning classifiers (extreme gradient boosting and random forest) with feature-importance outputs. **Results:** In a case study using anti-CD3/anti-CD28-stimulated peripheral blood mononuclear cell cytokine profiles from subjects with prediabetes at 20 and 72 h (33 samples per time point), CytokineProfile supported same-dataset comparison with MetaboAnalyst and CytokineExplore, generated consistent group separation in supervised analyses, and provided complementary volcano, dual-flashlight, and machine-learning-based feature results. Benchmarking on datasets up to 1,000 samples and 100 cytokines showed practical runtime and memory usage for representative workflows. **Conclusion:** CytokineProfile provides a reproducible, user-friendly platform integrating quality control, statistical analysis, visualization, and machine learning. The tool streamlines cytokine biomarker discovery and interpretation, supporting rigorous analysis of complex datasets.

## Graphical Abstract

**Figure:**
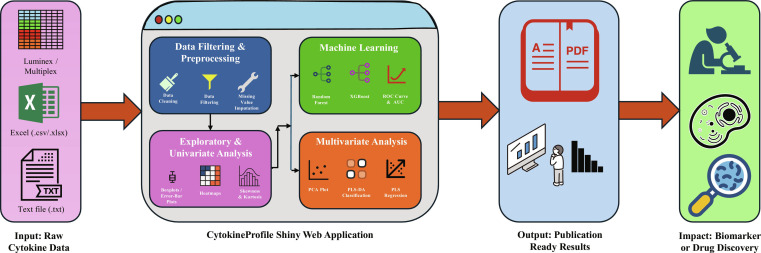

## Introduction

Cytokines are low-molecular-weight proteins that are secreted by a variety of cells, especially immune cells such as macrophages and lymphocytes. During immune response, cytokines act as messengers for immune cells. The response by immune cells depends upon which structures and cytokines stimulate them [1]. This makes cytokine profiling suitable as a biomarker not only for diseases directly impacting the immune system (i.e., infectious diseases) but also for noncommunicable diseases with altered immune function (e.g., diabetes). The cytokine profile can predict disease progression and clinical outcomes [2], distinguish the pathogenesis of a disease [3,4], and explore new biomarkers for treatment development [5,6].

High-dimensional cytokine studies benefit from workflows that are both statistically sound and approachable to researchers across various roles. Not all researchers are experts at programming, which can prove to be a barrier as one would have to conduct varied and extensive data management and manipulation to perform statistical analysis. To overcome this barrier, web applications must provide a user-friendly approach for researchers to conduct their analysis and obtain manuscript-ready results without having any extensive programming experience.

Several web applications exist, but they all come with their respective limitations as the broad use is generally for metabolomics data or general omics workflows rather than for cytokine-focused study design and interpretation. CytokineExplore (https://cytokine-web.onrender.com), an online-only application, offers some statistical analysis capabilities like boxplots, histograms, principal component analysis (PCA), and partial least squares discriminant analysis (PLS-DA), enabling researchers to explore their cytokine data [7]. However, CytokineExplore lacks essential features for predicting cytokine profiles most relevant for disease, including data quality assessment, effect size calculation, and transformations based on skewness and kurtosis. Similarly, other analytical tools designed primarily for metabolomics, such as MetaboAnalyst [8,9] and Qlucore Omics Explorer (https://qlucore.com/qlucore-omics-explorer), require substantial adaptation for cytokine analysis and often do not fully meet the unique requirements of cytokine profiling. These limitations emphasize the need for a dedicated software tool capable of specialized data analysis and visualization tailored for cytokine profiling.

To address these needs, we introduce CytokineProfile, an R Shiny web application and a novel analytical tool specifically designed for cytokine profiling. The tool offers a comprehensive pipeline for data preprocessing, quality control, exploratory analysis, group comparison, cytokine identification, and disease biomarker discovery. CytokineProfile is built on the CytoProfile R package [10] and is intended to offer an accessible and user-friendly tool for users with minimal statistical and programming background.

In this paper, we compare CytokineProfile’s functionalities with those of CytokineExplore and MetaboAnalyst’s one-factor statistical analysis methods using same-dataset comparison, highlighting the enhancements and specialized capabilities that CytokineProfile brings to cytokine data analysis. We focus particularly on the integration of machine learning (ML)—such as extreme gradient boosting (XGBoost) [11], random forest (RF) [12,13], and sparse PLS-DA (sPLS-DA) [14]—and effect size within an accessible, free-to-use application built using the R programming language (version 4.5.3) [15]. This comparison aims to show CytokineProfile’s contributions to advancing cytokine profiling and its likely impact on both research and clinical applications. We chose not to include Qlucore Omics Explorer in the overall comparison as it is paid software, and we did not obtain a license to conduct analyses and generate results for the comparisons.

## Application Overview

**Fig. 1.**
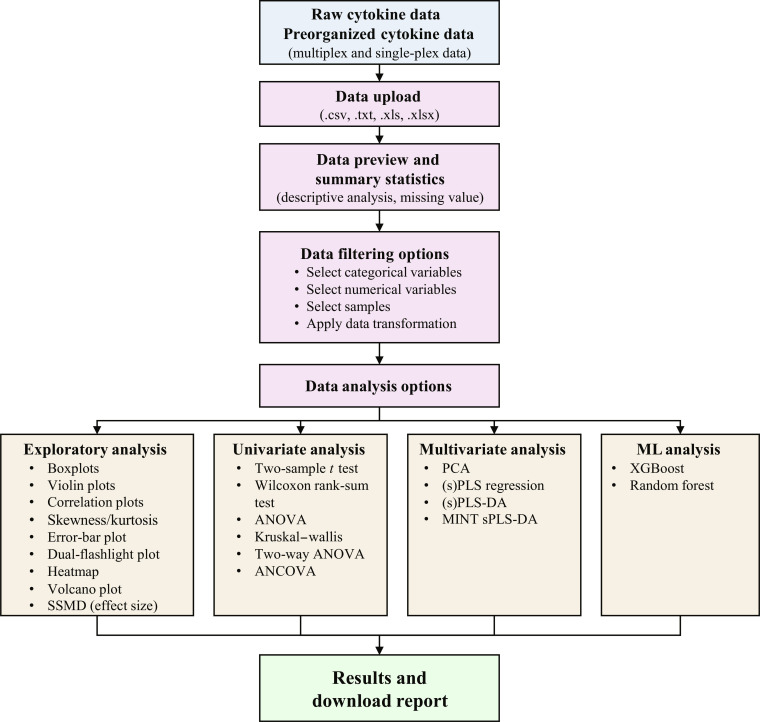
CytokineProfile application conceptual workflow from uploading data to obtaining results. Overview of the CytokineProfile analysis pipeline from data upload to result export. The application accepts raw or preorganized cytokine data tables and supports data preview and summary statistics, filtering and preprocessing, exploratory and univariate analyses, multivariate modeling, machine-learning classification, and export of organized results.

The application presents a very simple and easy-to-follow workflow as seen in Fig. 1. Below is a brief description of what to expect during the use of the application.

Data upload: CytokineProfile starts off with a simple data upload. Users may extract their multiplex or single-plex cytokine data (e.g., from their Luminex or Meso Scale Discovery assay) in “.xls” or “.xlsx” format. Users can choose to upload either the raw data or the preorganized data file with sample information such as groups, treatments, and time points. Users can upload data in one of the following accepted formats: “.csv”, “.txt”, “.xls”, and “.xlsx”.

Data editor: Once they upload a file, they can use the “Data Editor” feature to remove and add information to the dataset without affecting the original file. This is especially practical to organize raw data files. The data are also automatically formatted when progressing to step 2 by handling cytokine concentration values marked with “*” for values below the range and any out-of-range (OOR) values. This streamlines the process for uploading the necessary data to analyze after receiving an Excel file from the Bio-Plex software.

Data preview and summary statistics: On the same page as the data upload, the application provides options to quickly preview the data and see summary statistics to help guide in data preprocessing. Users can easily notice if there is a missing value in their data. For missing values in the dataset, in step 2, there is an option to handle missing values using several different methods depending on the user’s choice and needs, which includes imputation by mean, median, mode, and *k*-nearest neighbors (kNNs).

Missing-data imputation and OOR handling: CytokineProfile supports mean, median, mode, and kNN imputation. Mean and median provide simple options when missingness is limited, whereas kNN may better preserve the multivariate structure when correlated samples or cytokines are present. Because any imputation strategy can affect variability, *P* values, clustering, and multivariate modeling, users should interpret downstream results cautiously when missingness is substantial or differs by group. CytokineProfile also detects assay values marked as below or above range and applies rule-based replacements while reporting the affected variables to the user. Detailed method-specific guidance for choosing an imputation strategy and the exact rules used for OOR value handling are provided in Note S1.

Data analysis options: Once the data are uploaded, the app organizes the available methods into 4 panels:

• Univariate tests: In addition to the exploratory methods, the application has options to conduct 2 different univariate tests.
  o Analysis of variance (ANOVA)/Kruskal–Wallis to test mean differences across ≥3 groups.
  o Welch 2-sample *t* test/Wilcoxon rank-sum test to assess differences between 2 groups.
  o Two-way ANOVA and analysis of covariance for more complex study designs to determine differences between given groups, factors, and covariates.
• Exploratory analysis: Seven different methods for exploring datasets.
  o Boxplots to summarize distributions and outliers with supported stratification-based boxplots.
  o Violin plots for another variation of visualization to summarize distribution and outliers, supported with stratification.
  o Correlation plots to assess correlation between variables of interest.
  o Skewness and kurtosis to assess symmetry and tail weight before inferential steps.
  o Error bar plots pairing group differences with *P* values/effect sizes.
  o Dual-flashlight plots [16,17] to examine average log_2_ fold change versus strictly standardized mean differences (SSMDs), aiding the prioritization of variables.
  o Volcano plots showing log fold change and statistical significance and highlighting candidate variables for follow-up analysis.
• Multivariate analysis: The application also provides multivariate analysis methods adopted from the mixOmics R package [14,18].
  o PCA reduces dimensionality and uncovers patterns [19], for structure/pattern discovery.
  o (s)PLS regression for supervised modeling relationship with a continuous outcome variable from a high-dimensional dataset of predictor variables. The sparse version performs variable selection to identify a small subset of the most influential predictors that explain the outcome.
  o sPLS-DA for supervised discrimination and biomarker discovery. Users can apply multilevel analysis for data with repeated measurements (e.g., pre- vs. posttreatment) and batch-effect correction using the *z*-scoring method.
  o Multivariate integration sparse partial least squares discriminant analysis (MINT sPLS-DA) to model batch-stratified cohorts while retaining discrimination.
• ML: Lastly, we have also implemented 2 ML functions to provide more robust methods for classification.
  o XGBoost from the xgboost R package [11] for binary/multiclass tasks with feature importance.
  o RF using the randomForest R package [12] with optional receiver operating characteristic (ROC)/area under the curve (AUC) for binary setups and ranked features.
• Results: Once an analysis is finished, all outputs are rendered in tabbed panes so figures/tables from different steps remain organized and easy to export.

### Software implementation, reproducibility, and computational performance

CytokineProfile is implemented as an R Shiny web application with a reactive user interface and an analysis backend built on the CytoProfile package together with established R libraries for visualization, multivariate analysis, and ML. Major dependencies include “mixOmics” for multivariate modeling, “randomForest” for ensemble classification, “xgboost” for gradient-boosted tree models, and supporting packages for statistical testing, post hoc contrasts, and graphics. To improve reproducibility, stochastic components use a fixed default random seed of “123456”, the source code is maintained in a version-controlled GitHub repository, package versions are reported in Table ST1, and core functions are verified using unit tests implemented with the “testthat” [20] and “shinytest2” [21] framework. Analyses can also be reproduced offline by installing the application as an R package locally and rerunning the same parameters used in the web interface.

To assess computational behavior under realistic usage conditions, we performed benchmarking on simulated cytokine datasets of increasing size, including datasets up to 1,000 samples and 100 cytokines. For each dataset size, we recorded wall-clock runtime and peak memory usage for representative workflow components, including data upload/preprocessing, exploratory visualization, sPLS-DA, RF, and XGBoost. Benchmarks were conducted on a university-provided Windows laptop using R 4.5.3, and median runtime and peak memory are summarized in Fig. S1. These benchmarks were conducted on the underlying analysis functions that power the application rather than as full end-to-end timing of interactive Shiny sessions. Accordingly, the reported runtime and memory values reflect the computational cost of the core analytical routines under the local environment, whereas the end-to-end responsiveness of the web application also depends on deployment resources, browser rendering, and concurrent usage.

Because interactive performance depends in part on deployment resources, these values should be interpreted as empirical performance for the current deployment rather than universal software limits. The public instance is hosted on Posit Connect Cloud, where application memory, central processing unit allocation, worker settings, and connection limits are deployment and plan dependent.

### Multivariate and ML evaluation

For the RF and XGBoost modules, all reported performance metrics such as accuracy, sensitivity, specificity, confusion matrix, and AUC for binary classification are computed exclusively on an independent held-out test set. This test set is created by random splitting by the user inputting a value between 0.1 and 0.9 for the argument “Train Fraction” in both RF and XGBoost. The training set is used exclusively for model fitting. Additionally, both methods provide optional cross-validation (CV) through different methods. XGBoost uses *k*-fold CV that only runs on the training partition. CV results are reported in a separate section of the summary output and are intended to supplement, not replace, the held-out test evaluation. For RF, CV is optionally available for feature-selection CV. This runs on the training data only and is intended to guide optimal feature count selection and not to report final predictive performance.

### Case study dataset and analysis workflow

To demonstrate CytokineProfile on real data and to complement the feature-based comparison in Table 1, we used a cytokine dataset derived from Pugh et al. [6]. The cross-sectional study recruited individuals with overweight/obesity and normal glycemic control or prediabetes (PreT2D) or type 2 diabetes (T2D). Peripheral blood mononuclear cells (PBMCs) were isolated and then cultured in 24-well plates (CellStar) at 500,000 cells/well. Cells were stimulated with anti-CD3/anti-CD-28 Dynabeads (Gibco) for 20 to 72 h. A total of 25 cytokines were measured from the supernatant of the stimulated cells using a 25-plex Th17 magnetic bead kit (Millipore Sigma), and signals were detected using the Luminex platform. The Bio-Rad FLEXMAP 3D with Luminex xPONENT version 4.2 and Bio-Plex Manager (Bio-Rad) software were used to perform data acquisition and subsequent concentration calculations according to the manufacturer’s instructions. Values with bead counts below 50 were excluded.

**Table 1.** Documented feature availability across CytokineProfile and existing tools

Feature	Software comparison		
	CytokineProfile	CytokineExplore	MetaboAnalyst
General information			
Website	https://shinyinfo.cytokineprofile.org/	https://cytokine-web.onrender.com/	https://www.metaboanalyst.ca/home.xhtml
Latest version	0.0.1 (release on publication) 0.0.0.9000 (development version)	N/A	6.0
Release date	TBD	2020 July 31	May 2009
Primary purpose	Cytokine profiling	Online exploratory statistical analysis tool for cytokine concentration data	Metabolomic data analysis
Price	Free	Free	Free
Data handling			
Input data format	Excel (.csv and .xlsx), .txt	Excel (.xlsx)	.csv, .txt, .zip, NMR/MS spectra
Data preprocessing	Data transformation, data scaling	Data transformation, sample normalization	Data transformation, data scaling
Data management	Website upload	Website upload	Website upload
Analysis features			
Statistical analysis	ANOVA, *t* tests, Wilcoxon test, PCA, sPLS-DA, (s)PLS regression, MINT sPLS-DA, XGBoost, random forest	PCA, PLS-DA, Mann–Whitney *U* test, summary statistics	ANOVA, *t* tests, PCA, (s)PLS-DA, random forest, support vector machine
Cross-validation	LOOCV, *k*-fold	N/A	LOOCV, *k*-fold
Customizability	Extensive (ellipses, shaded background, confusion matrices)	N/A	Limited
Visualizations			
Heatmaps	Yes	Yes	Yes
Volcano plots	Yes	No	Yes
3D plots	Yes	No	Yes
Loading plot	Yes	Yes	Yes
Boxplots	Yes	Yes	Yes
Error bar plot	Yes	No	No
Effect size (SSMD)	Yes	No	No
Dual-flashlight plot	Yes	No	No
Skewness and kurtosis plots	Yes	No	No
Export file types	PDF (comprehensive with all plots in one file)	PNG (limited to one image per file)	PNG (limited to one image per file)

N/A, not available; NMR/MS, nuclear magnetic resonance/mass spectrometry; ANOVA, analysis of variance; PCA, principal component analysis; sPLS-DA, sparse partial least squares discriminant analysis; MINT, multivariate integration; XGBoost, extreme gradient boosting; PLS-DA, partial least squares discriminant analysis; LOOCV, leave-one-out cross-validation; 3D, 3-dimensional; SSMD, strictly standardized mean difference

For the analyses, we filtered the dataset to include only subjects with PreT2D under the treatment of anti-CD3/anti-CD28 beads to determine how the cytokine profiles change between the 2 time points 20 and 72 h. This provided us with a sample size of 33 per time point. The shared comparison used 12 retained cytokines: IL-5, GM-CSF, IFN-γ, TNF-β, IL-27, IL-17A, IL-31, CCL-20/MIP-3A, IL-9, IL-13, IL-21, and IL-17F. All tools used log_2_ transformation for normalization on all cytokines. Because direct cross-tool comparison was limited to overlapping analyses and exportable outputs, we compared (s)PLS-DA across CytokineProfile, MetaboAnalyst, and CytokineExplore, while Volcano and RF comparisons were restricted to CytokineProfile and MetaboAnalyst. Parameters to reproduce the figures are available in Note S2.

## Comparisons of CytokineProfile with Existing Sources

### General comparison

Multiple tools can be used to analyze cytokine profiling data, but they differ in input requirements, supported workflows, and degree of cytokine-specific tailoring. Table 1 is presented as a summary of documented feature availability and workflow scope for cytokine data analysis.

When comparing the documented capabilities of CytokineProfile to those of established tools like MetaboAnalyst and CytokineExplore (see Table 1), several differences in workflow scope and supported outputs for our Shiny application emerge, especially in cytokine profiling. First, MetaboAnalyst and CytokineExplore require specific data formatting. MetaboAnalyst, which targets a broad set of metabolomics workflow, is unable to work directly with cytokine data derived from replicates without any reformatting. MetaboAnalyst requires the first column to be related to the sample, and the sample IDs need to be unique (e.g., duplicated sample names are not allowed). Additionally, MetaboAnalyst assumes that the second column of the uploaded Excel file consists of the grouping information. The same limitations also apply to CytokineExplore, which imposes strict formatting requirements. CytokineExplore requires the first column to contain group names followed by cytokine concentration values. This provides more work for the users to format their data prior to conducting analyses. Thus, it can limit usability for those with data in different formats. In addition, despite being specifically designed for cytokine data analysis, CytokineExplore has limitations on analysis options. The tool uses a tiered approach to results and visualizations. The analysis results are defined by the group provided, not by the users’ choice. For example, if users provide only one group, it will automatically generate PCA plots, histograms, boxplots, and correlation plots only. The PLS-DA approach, which we have used extensively and productively in cytokine profiling [4,22,23], can be generated only if 2 groups are provided. This may restrict analytical depth. In addition, CytokineExplore imposes practical constraints when larger cytokine panels are analyzed. Although figures can still be generated when many cytokines are included, the application does not reliably support download of the corresponding analysis outputs in those higher-dimensional settings. This limited export functionality, together with the requirement for exactly 2 groups to access PLS-DA, affected the scope of the same-dataset comparison and contributed to restricted 20-h versus 72-h time points of subject with the PreT2D subset.

In contrast, CytokineProfile is specifically designed for cytokine data analysis as it leads users through a guided 4-step wizard: upload/preview (step 1), filtering and variable selection (step 2), and method-specific analysis options (step 4), each with built-in explanations of parameters (Fig. S2A to D). The data formatting in CytokineProfile is flexible. Users can upload raw data and edit them directly in the application. Importantly, it provides various visualizations and integrated statistical and ML methods designed to enhance interpretability for cytokine data.

**Fig. 2.**
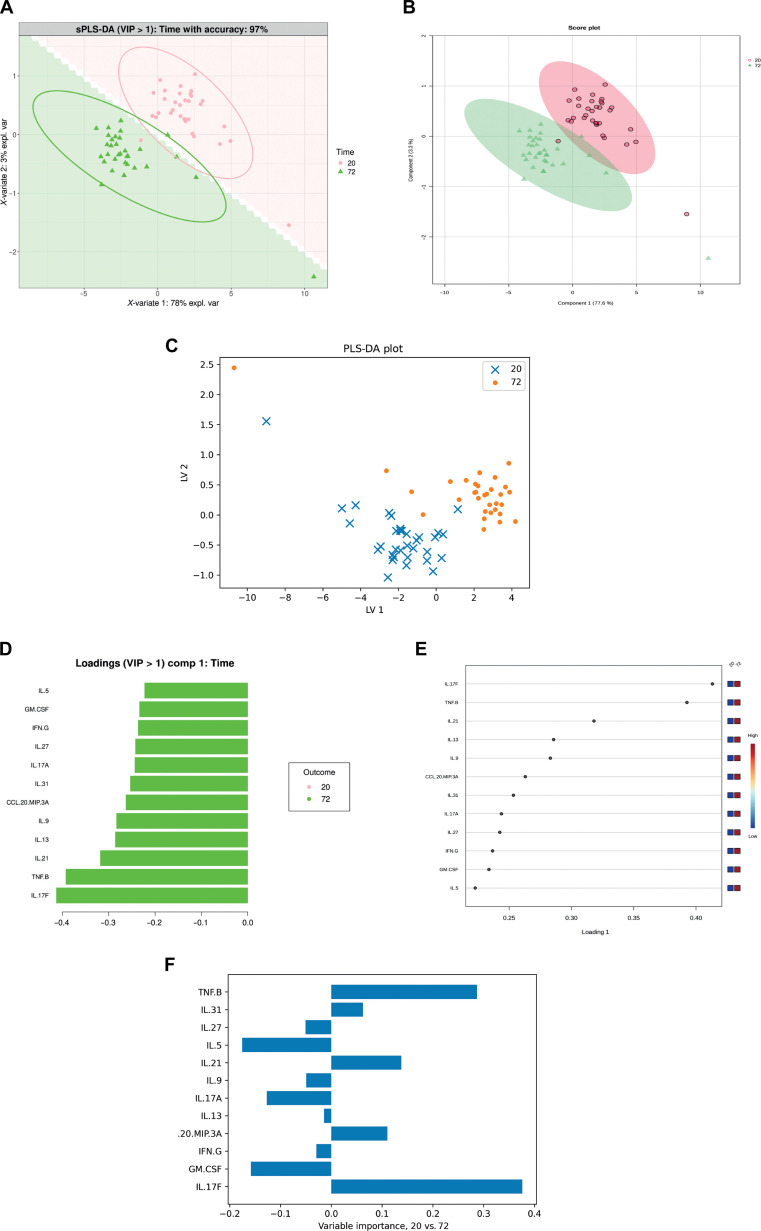
Same-dataset supervised-analysis comparison across CytokineProfile, MetaboAnalyst, and CytokineExplore for subjects with PreT2D under CD3/CD28 20 h versus 72 h comparison. All tools were applied to the same filtered subset of anti-CD3/anti-CD28-stimulated peripheral blood mononuclear cell (PBMC) supernatants from subjects with PreT2D, using the retained cytokines mentioned and log_2_-transformed cytokine concentrations. (A) CytokineProfile sparse partial least squares discriminant analysis (sPLS-DA) score plot using variable importance in projection (VIP) > 1 features, with shaded prediction background and 95% confidence ellipses. (B) MetaboAnalyst score plot for the same 2-class comparison. (C) CytokineExplore partial least squares discriminant analysis (PLS-DA) score plot for the same samples and cytokines. (D) CytokineProfile signed loadings for component 1 among the retained VIP > 1 cytokines. (E) MetaboAnalyst feature-contribution display for the same comparison. (F) CytokineExplore variable-importance summary for the 20 h versus 72 h comparison. Together, these panels show that the compared tools identify broadly similar group structures while differing in downstream interpretive outputs and visualization flexibility. Ellipses represent 95% confidence regions where shown.

Immune responses are dynamic and context dependent. Thus, understanding how immune cells contribute to disease progression is crucial for drug discovery. Here, we analyzed a cytokine dataset from a cross-sectional study by Pugh et al. [6] using CytokineProfile, MetaboAnalyst, and CytokineExplore (Fig. 2). For the purpose of the comparison between CytokineProfile and the existing tools, we analyzed only the PreT2D samples that had been stimulated with anti-CD3/anti-CD28 beads for 20 and 72 h to determine the effects of stimulation time on cytokine profiles in response to T-cell-targeted stimulation. All tools predicted that the cytokine profile induced by longer stimulation time differs from that of the shorter stimulation time (Fig. 2A to C). Next, we wanted to identify which cytokines differentiate between the 2 time points. The loading plots generated by CytokineProfile and MetaboAnalyst show the same cytokines of importance (Fig. 2D and E). Importantly, all cytokines related to effector T cells were distinguished for the 72-h stimulation, suggesting that a longer stimulation time leads to more active effector T cells. Although the variable-importance summary generated by CytokineExplore shows a similar pattern (Fig. 2F), the absence of group representation by color makes it difficult to identify which time point is associated with the cytokine of importance. Because the sPLS-DA model is prone to overfitting, we evaluated the model by performing CV using the leave-one-out method and calculating the ROC and AUC (Fig. S3C to E). The model has CV accuracies of approximately 92% and 88% based on CytokineProfile and MetaboAnalyst’s leave-one-out CV, respectively, indicating that the separation between 20- and 72-h stimulation times is not due to overfitting. The ROC curve shows an almost perfect classification with an AUC of 0.9954 complementing the model’s performance determined by CV. Despite their important function in evaluating the sPLS-DA model, ROC with AUC for sPLS-DA is available only in CytokineProfile.

Another set of features that further distinguishes CytokineProfile from MetaboAnalyst and CytokineExplore supports extensive customization in its (s)PLS-DA, allowing users to incorporate ellipses that draw a 95% confidence region around the data points or shaded prediction backgrounds for enhanced display of group discrimination. Shaded prediction background is computationally heavy and increases the time to generate outputs; however, the ellipses also provide a way to see the discrimination from sPLS-DA. With little to no overlap in ellipses, the model shows moderate to good discrimination; however, with major overlap, the model shows that the groups in comparison are unable to be distinguished. Within the sPLS-DA, CytokineProfile offers repeated-measures (multilevel) analysis that is suitable for study with multiple data points such as in randomized control trials (i.e., pre- and posttreatment). Furthermore, our application allows users to perform batch effects correction by *z*-scoring method and MINT sPLS-DA, an exclusive method for accounting multiple batches while performing classification. This feature is crucial to limit the batch effects resulting from measuring cytokine levels in multiple plates/batches. Users can also plot individual point names; hence, they can identify which samples fall outside the 95% confidence region drawn by ellipses. Using this feature, users can check the sample information to investigate if there is potential bias or confounding factors (e.g., seasons, economic status, and medications). The sPLS-DA function also has an option to generate confusion matrices to illustrate sPLS-DA classification [24], surpassing the limited customization options offered by MetaboAnalyst and CytokineExplore. Lastly, the sPLS-DA function provides both static and interactive 3-dimensional score plots. Points represent samples mapped to the first 3 latent components (components 1 to 3). The static 3-dimensional snapshot is included in the exported files, while the in-app interactive view supports rotation and zoom and shows sample identifiers on hover using plotly [25]. When a multilevel (repeated-measures) design is selected, samples are annotated with subject IDs to aid inspection of within-subject trajectories and potential outliers. Extended CytokineProfile sPLS-DA figures, including the in-app result panel, variable importance in projection scores, ROC and AUC, and CV summary, are shown in Fig. S3.

**Fig. 3.**
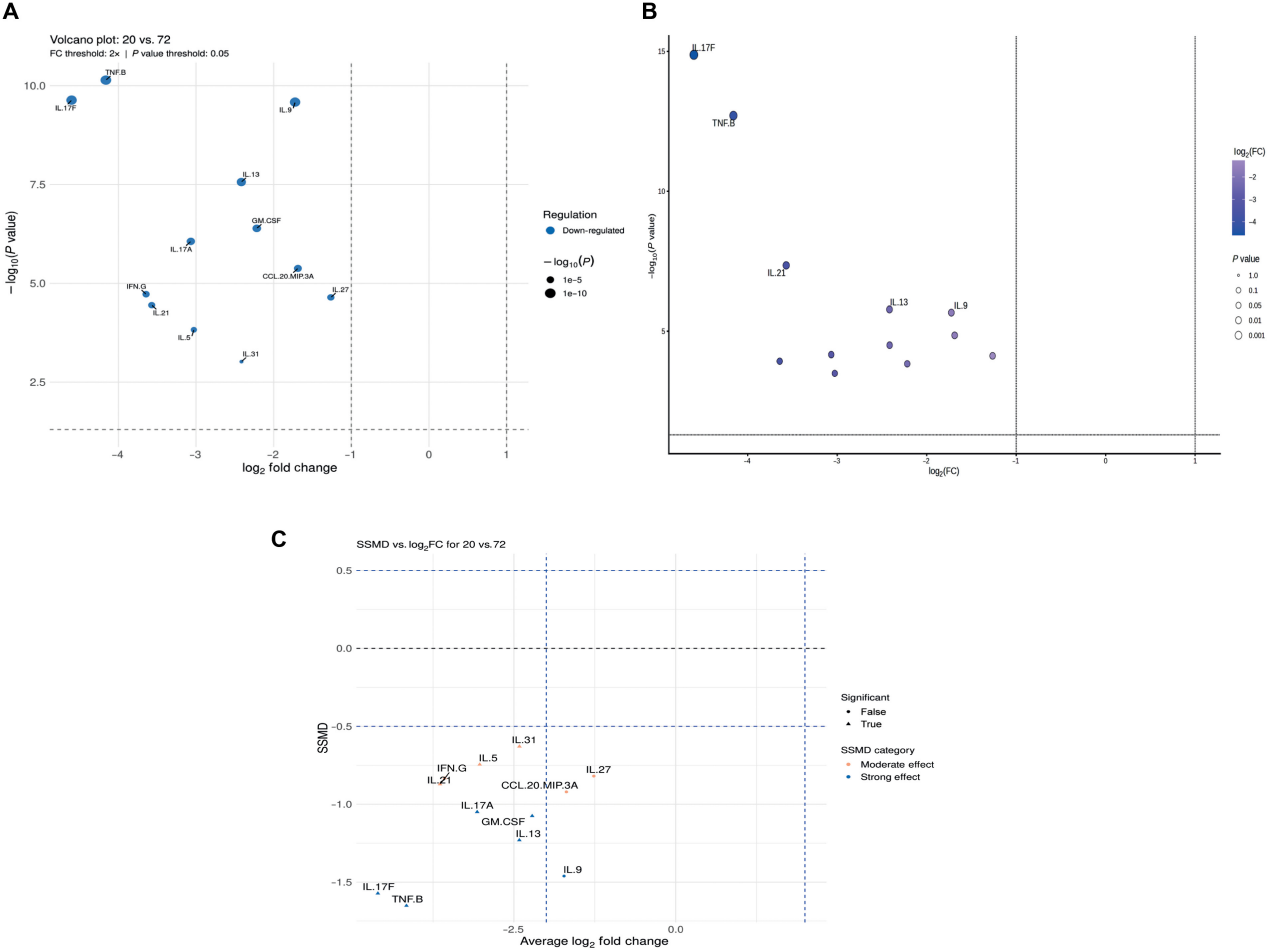
Differential-analysis outputs for the PreT2D 20 h versus 72 h comparison in CytokineProfile. (A) CytokineProfile volcano plot showing log_2_ fold change versus statistical significance for the retained cytokines in the 20 h versus 72 h comparison. Vertical and horizontal threshold lines indicate the selected log_2_ fold change and *P* value cutoffs. (B) MetaboAnalyst volcano plot showing the same comparison. (C) Dual-flashlight plot showing average log_2_ fold change versus strictly standardized mean difference (SSMD) for the same cytokines. This plot complements the volcano plot by emphasizing effect size in addition to directional change, helping distinguish cytokines with a stronger biological magnitude from those prioritized only by *P* value. Cytokines passing the selected thresholds are labeled in each panel.

To illustrate biologically oriented differential analysis, CytokineProfile provides additional methods to determine significance and effect size of cytokines. Figure 3A shows the volcano plot compared with MetaboAnalyst’s volcano plot in Fig. 3B and the dual-flashlight plot for the PreT2D 20 h versus 72 h comparison in Fig. 3C. The volcano plot emphasizes statistical significance together with fold change, whereas the dual-flashlight plot pairs log_2_ fold change with SSMD to highlight cytokines with meaningful effect sizes even when *P* values are more modest. This combined view supports prioritization of cytokines for follow-up rather than relying only on significance ranking. Both volcano plots show that cytokines associated with effector T cells were down-regulated in 20-h stimulation time (up-regulated in 72-h stimulation time), reinforcing the idea that a longer stimulation time increases effector T cell’s activity. The dual-flashlight plot points out Th1/Th17 (subsets of effector T cells) signature cytokines (IFN-γ, TNF-β, IL-17A, IL-17F, and IL-21) have a consistent and strong effect in differentiating the 2 time points. This narrows down which T cell subsets that are more active in sustained stimulation. Additionally, it emphasizes that when there is sustained T-cell-targeted stimulation, the PBMCs of individuals with obesity and prediabetes may shift toward the “proinflammatory” response and lack of regulatory response.

**Fig. 4.**
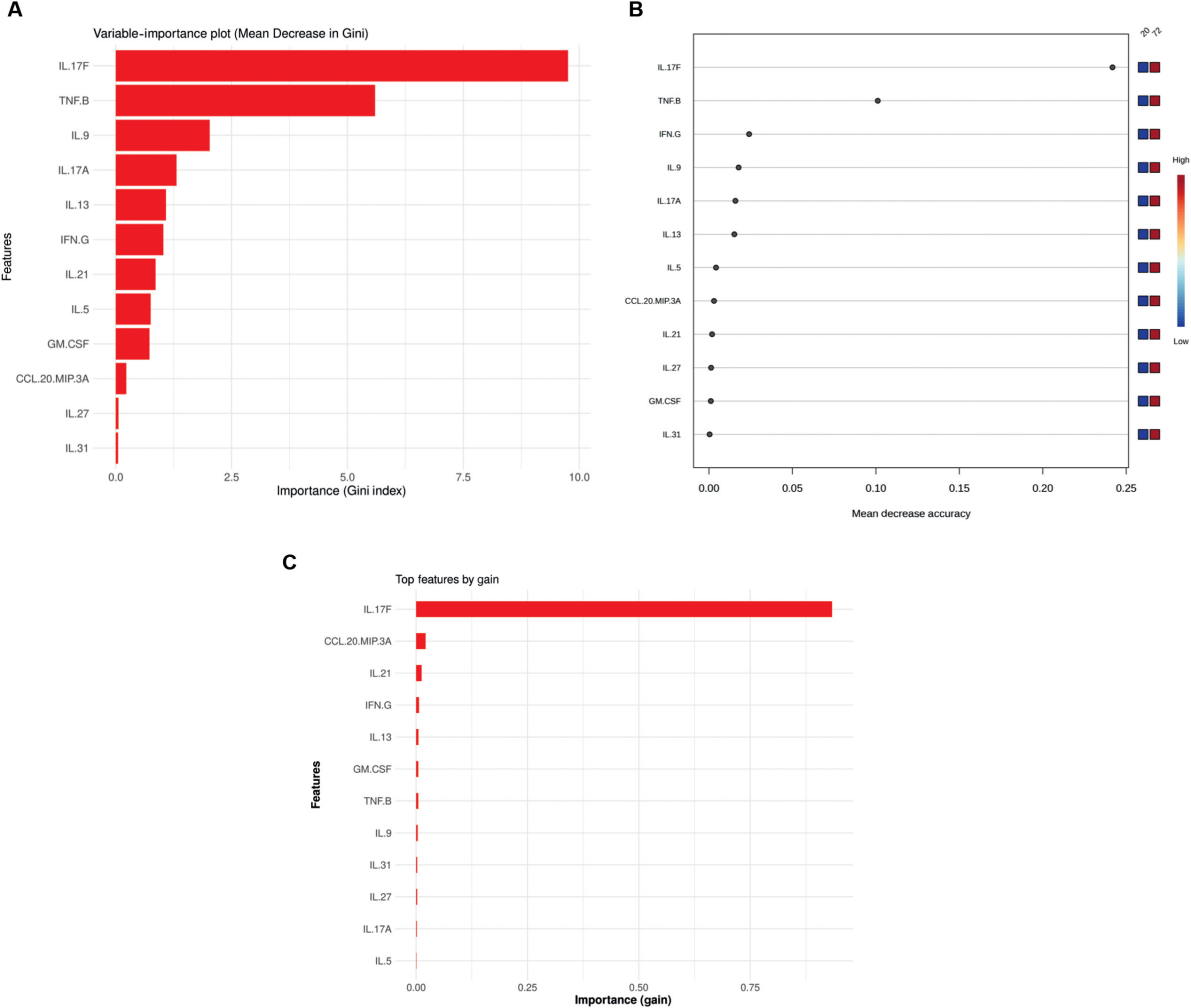
Machine-learning feature-prioritization outputs for the PreT2D 20 h versus 72 h comparison. (A) CytokineProfile random forest variable-importance ranking based on Mean Decrease in Gini. (B) Comparator random forest feature-importance summary for the same dataset. (C) CytokineProfile extreme gradient boosting (XGBoost) feature ranking based on gain. These panels illustrate how discriminatory cytokines are prioritized across complementary machine-learning workflows using the same filtered cytokine subset.

The integration of RF and XGBoost in CytokineProfile further enhances its capabilities. These methods improve classification and prediction, adeptly handling both small and large datasets while prioritizing important features. They also enhance the ability to classify disease states and accurately identify biomarkers. To further assess discriminatory cytokines in the same 20 h versus 72 h comparison, we compared feature-importance outputs from RF to MetaboAnalyst’s results. CytokineProfile generates clear feature-importance plots, allowing researchers to identify critical cytokines for group separation—RF using Mean Decrease in Gini (Fig. 4A) and XGBoost calculating gain (Fig. 4C), offering complementary insights alongside model performance with ROC curves and AUC values as seen in Fig. S4A to D. Here, we identified IL-17F and TNF-β as the 2 top cytokines differentiate the 72-h stimulation to the 20-h stimulation time. This highlights that sustained T-cell-targeted stimulation favors the production of Th1/Th17 signature cytokines in individuals with obesity and prediabetes.

Finally, CytokineProfile’s ability to generate results in various formats (.png, .tiff, .pdf, .svg, and .jpeg) adds important value for researchers seeking high-resolution figures and presentation-ready output. Additionally, the tool provides font customizability settings to adjust text sizes in axes and legends to ensure best readability in the output figures. These attributes make CytokineProfile a useful cytokine-focused analysis platform for cytokine research, enhancing both the efficiency and depth of data analysis in immunological studies.

### User-friendliness comparisons of web-based versions

While user-friendliness is subjective, we believe that CytokineProfile provides a much more streamlined approach by utilizing a wizard-style step process. Tutorials, including videos on how to use the application and result examples, are also provided in the application. This will help users navigate the application and understand their results better. The workflow is similar in a broader context to those of other tools with upload of data, choosing variables to keep in analysis, and choosing the types of analysis with subcategories of specific methods to utilize to generate results. However, CytokineProfile does more than the tools we have compared to. To start with, when a dataset is uploaded, CytokineProfile provides an option of previewing the data and summary statistics including but not limited to mean, 25th percentile, median, and 75th percentile values and provides an option to view the dataset. Additionally, the application supports a “Data Editor”, which opens up a new modal allowing users to edit column names, create new columns and insert values, and delete either rows or columns (Fig. S2A and B).

As mentioned previously, CytokineExplore uses a tiered approach where the user must define a set number of grouping variables to achieve specific results and continuously change parameters to achieve their goal. CytokineProfile, on the other hand, provides filtering options in step 2 of the process, giving a direct option to select specific variables and filter categorical variables while still allowing them to conduct different statistical tests (Fig. S2C). Additionally, the user also has the option to explicitly delete rows that will not be used for the analysis and can be restored either all at once or for a selected subset of rows.

Lastly, when the user selects a method to start their analysis, for example, boxplots, sPLS-DA, or volcano plots, CytokineProfile provides users with the arguments for further customization of visuals (e.g., selecting plotting symbols and colors and changing the limits of axes and threshold values), which are dependent on the methods. The CytokineProfile app also provides helper labels for each argument with either a question mark or an exclamation mark symbol that gives users extra information on the functionality of the arguments for users to understand the arguments if they are unfamiliar with a specific argument (Fig. S2D). In contrast, CytokineExplore and MetaboAnalyst do not surface method-specific arguments with inline explanations to the same extent, making them less accessible to users with minimal statistical background.

## Discussion and Conclusion

High-dimensional cytokine profiling is increasingly used for biomarker discovery and disease characterization, particularly in immunology and metabolic disorders. However, existing analytical platforms are largely designed for metabolomics or general omics data and often require substantial data reformatting, fragmented preprocessing, and disjoint analytical workflows. For example, current workflows often force investigators to manually reformat assay outputs, handle quality control and batch effects by hand, and stitch together separate statistical and ML steps. This effectively puts a new high technical barrier in front of researchers collecting cytokine profiling data. CytokineProfile was developed to address exactly that barrier: it is a cytokine-focused platform that accepts common assay outputs, standardizes preprocessing and filtering, and then delivers statistically rigorous and biologically interpretable results—all within one environment.

CytokineProfile integrates multiple analytical layers within a unified framework. These include exploratory visualization, normality-informed univariate testing, multivariate modeling (PCA and sPLS-DA with multilevel and MINT extensions), and supervised ML approaches (RF and XGBoost). Importantly, the results demonstrate that this integrated workflow enables consistent identification of group separation and key cytokines in the same-dataset comparison with MetaboAnalyst and CytokineExplore while additionally providing complementary outputs such as ROC/AUC evaluation, dual-flashlight plots, and flexible feature-importance summaries. These results support the utility of CytokineProfile as both an exploratory and hypothesis-generating tool for cytokine biomarker discovery.

A distinguishing feature of CytokineProfile is the incorporation of effect-size-driven analysis through the dual-flashlight plot [16,17,26], which jointly visualizes both log_2_ fold changes (log_2_FC) and SSMD [26–28]. The dual-flashlight plot uses SSMD rather than *P* values on the *y*-axis. This distinction is critical because *P* values conflate effect size with sample size—they shrink toward zero with a large *n*, even when mean differences are negligible. In contrast, SSMD isolates effect size and converges to its true population value regardless of sample size [26,29]. Moreover, SSMD has a direct mathematical relationship with area under the ROC curve, a widely adopted metric for performance evaluation in ML [30]. As shown in the results, this approach complements traditional volcano plots by prioritizing cytokines with meaningful biological effect sizes rather than relying solely on statistical significance.

The same-dataset comparison further highlights practical advantages of CytokineProfile over existing tools. Unlike MetaboAnalyst and CytokineExplore, which impose strict input formatting requirements and limited customization, CytokineProfile supports flexible data structures, integrated preprocessing (including missing-value handling and OOR adjustment), and user-guided parameter selection. Additionally, CytokineProfile extends beyond existing tools by enabling multilevel modeling, batch-effect correction, and comprehensive ML evaluation within a single interface. These features are particularly important for cytokine studies involving repeated measures, heterogeneous cohorts, or high-dimensional panels, as demonstrated in the PBMC stimulation case study.

Despite these strengths, several limitations should be considered. First, although CytokineProfile is intended as an integrated exploratory and analytical platform, interactive performance depends on both dataset size and deployment resources; therefore, practical capacity limits should be interpreted empirically rather than as universal software boundaries. In our benchmarking runs, representative workflows were evaluated on datasets up to 1,000 samples and 100 cytokines, which define the largest dataset sizes systematically assessed in this study rather than a universal upper limit for the application. In addition, the reported benchmarking results were generated from the core analytical routines rather than full end-to-end web-interface sessions, so interactive latency in the deployed application may differ depending on hosting resources and user load. Second, the public instance is hosted on Posit Connect Cloud and should be viewed as a convenience deployment rather than the only supported environment. Long-term sustainability is supported by the publicly available source code and local installation pathway. Third, the cross-tool comparison in this study was restricted to overlapping analyses available across platforms and therefore should be interpreted as a practical same-dataset comparison rather than a comprehensive comparison of all features. Fourth, cytokine measurements remain influenced by assay platform, dynamic range, bead-count thresholds, and preprocessing decisions for OOR values, so study comparisons and downstream biological interpretation should be made cautiously. Finally, the current ML modules are best viewed as research-support tools rather than locked predictive systems, because performance estimates are based on held-out test splits and optional training set CV rather than a full nested-resampling or external-validation framework.

Future work will focus on extending the analytical capabilities of CytokineProfile, including incorporation of additional ML methods such as support vector machines [31,32] and advanced neural networks [33] as well as extending hyperparameter tuning for XGBoost. These methods are exceptionally skilled at capturing the complex, nonlinear relationships often present in high-dimensional biological data. By adding these models, CytokineProfile’s predictive power will be significantly improved, leading to more accurate biomarker identification and a deeper understanding of intricate disease mechanisms through cytokine analysis.

In summary, CytokineProfile integrates preprocessing, visualization, univariate testing, multivariate modeling, and ML modules within a cytokine-focused workflow. Compared to existing software (i.e., MetaboAnalyst and CytokineExplore), CytokineProfile offers flexibility of data formatting and comprehensive data analysis tailored to cytokine profiling. Additionally, our tool allows users to customize the visuals as needed. The tutorials and the argument pop-up feature guide users through the process, making high-dimensional data analyses more accessible to users with minimal statistical background. By facilitating precise data classification and feature selection, the tool strengthens our ability to understand immune responses, discover new biomarkers, and identify therapeutic targets. As a valuable new resource for the bioinformatics community, CytokineProfile is positioned to provide a practical resource for immunological studies and deepen the interpretive value of cytokine data.

## Data Availability

The analytic tool of CytokineProfile for users is currently hosted on Posit Connect Cloud and is available to access (https://shiny.cytokineprofile.org/). The source code is available at the GitHub page of the CytokineProfile application (https://github.com/ZhangLabUKY/CytokineProfileShinyApp). Tutorials on running the application locally for offline use and basic tutorials for analysis methods are also available on the CytokineProfile website (https://shinyinfo.cytokineprofile.org/articles/index.html). The case study dataset used for the manuscript figures and the parameter settings required to reproduce Figs. 2 to 4 and Figs. S1 to S4 are provided in the Supplementary Materials.

## Supplementary Materials

Supplementary 1 Case Study Dataset Figs. S1 to S4 Table ST1 Notes S1 and S2
