# Supplementary material for: CytokineProfile: An Integrated Web Tool for Cytokine Profiling Analysis
Source: Comput Struct Biotechnol J. 2026 May 7;35(1):0079. doi: 10.34133/csbj.0079 (PMC13150067; doi:10.34133/csbj.0079)
Supplement: Supplementary 1 — Case Study Dataset Figs. S1 to S4 Table ST1 Notes S1 and S2 [file csbj.0079.f1.zip › Supplementary.Information.docx]

**Supplementary Information and Figure Legends**

**Supplementary Note S1. Missing-data imputation and out-of-range handling**

**Choosing an Imputation Method for Missing Values:** Default behavior: The default behavior for the imputation step is to go with mean imputation, one of the most common approaches. When working with kNN imputation, the default number of neighbors is k = 5 and adjustable by user as they see fit.

How to choose an imputation method

- If missingness is low and you want a simple numeric fill, start with median for skewed cytokine data and mean for roughly symmetric data.
- If preserving multivariate structure matters, consider kNN instead of a simple fill.
- Use sample-wise kNN when you expect biologically similar samples or subjects.
- Use feature-wise kNN when correlated cytokines are expected and only numeric features are being imputed.
- If missingness is substantial or clearly differs by group, interpret downstream results more cautiously.

Method-by-method guidance

- Mean: numeric columns only. This is a simple baseline when missingness is limited and values are roughly symmetric. It can reduce variability and pull group summaries toward the center.
- Median: numeric columns only. This is often a better choice when cytokine values are skewed or outliers are present. It still compresses spread and can mute real group differences.
- Mode: categorical columns only in the current app. It is useful for labels or discrete annotations, not for continuous cytokine concentrations. It can over-represent the most common category.
- kNN (sample-wise): works across the selected columns using similar samples or rows and can handle mixed data. This is useful when biologically similar subjects are expected. It can blur separation between phenotypes or treatment groups if nearest neighbors come from different groups.
- kNN (feature-wise): numeric columns only. This method uses similar analytes or features across samples, which can be useful when correlated cytokines tend to move together. It can reinforce correlation patterns and overstate coordinated biology.

Bias and interpretation notes

Simple imputation methods can underestimate variability and affect p-values, clustering, and multivariate models in cytokine profiling studies. kNN usually preserves local structure better than mean or median imputation, but it still inserts modeled values rather than directly observed measurements.

**Handling of Out-Of-Range Values:** Cytokine profiling may be limited by the detection range of the assay kit. Cytokines reads from the assay platform are prepared using the Luminex softwares (e.g. Bio-Rad FLEXMAP 3D with Luminex xPONENT, Bio-Plex Manager (Bio-Rad)). The Bio-Plex Manager calculates the cytokine concentrations based on the standard curve constructed by the known concentration from a series of standards and fluorescence intensity (detected by FLEXMAP 3D). Depending on the samples, kit, and context (e.g. disease state), the concentration calculation may result in OOR values. When a file is uploaded that consists of columns with out-of-range values marked as OOR< (out-of-range below) or OOR> (out-of-range above), and the user clicks “save &use”, a new warning message is prompted that describes the handling of out-of-range as follows:

- Shows the total out-of-range values and numbers for above and below range.
- Shows affected columns, such as Cytokine 2, Cytokine 5, etc.
- OOR> value defined as: Values marked above range are replaced with the maximum value from the column plus one percent of that column's observed range.
- OOR< value defined as: Values marked below range are replaced with the column minimum plus ten percent of that minimum when the column minimum is positive.
- Additional safeguard for OOR<: If the column minimum is zero or negative, values marked below range are replaced with that minimum.

**Supplementary Note S2. Parameters Used in Generating Comparison Figures For Each Method and Software.**

The following parameters were set for sPLS-DA:

- Number of variables: 12
- Number of components: 2
- Ellipse/95% confidence region was enabled in both CytokineProfile and MetaboAnalyst.
- CytokineProfile additionally used shaded background prediction for better visualization of the classification.

Random Forest uses the following parameters:

- Number of Trees: 500
- Number of variables to split (mtry): 5
- Training fraction (only configurable in CytokineProfile): 0.7
- Seed fixed to 123456 on MetaboAnalyst to match the default seed fixed in CytokineProfile.

XGBoost uses the following parameters:

- Number of Rounds: 500
- Learning rate: 0.1
- Maximum Depth: 4
- Evaluation metric: auc
- Minimum loss reduction required to make a further partition on a leaf node of the tree: 0 (fixed internally)
- Minimum sum of instance weight: 1 (fixed internally)
- Subsample ratio of the training instance: 1 (fixed internally)
- Subsample ratio of columns when constructing each tree: 1 (fixed internally)

Volcano Plot Parameters:

- Log_2_ fold change threshold: 2
- P-value threshold: 0.05

Dual-flashlight Plot Parameters:

- Log_2_ fold change threshold: 2
- SSMD threshold: 0.5

**Figure S1. Computational benchmarking summary for CytokineProfile core analysis functions across dataset sizes and analysis modules.** (A) Benchmark of core CytokineProfile analytical functions showing median completion time in seconds and peak memory (RAM) usage on a simulated complete dataset. (B) Benchmark of core CytokineProfile analytical functions showing median completion time in seconds and peak memory (RAM) usage on a simulated data set with missing values and imputation.

**Figure S2. Step-by-step CytokineProfile interface for data upload, editing, filtering, and parameter entry.** (A) Step 1 data-upload interface showing accepted file formats together with data preview and summary-statistics tabs. (B) Data Editor pop-up used to modify imported tables without changing the original source file. (C) Step 2 filtering interface used to select categorical and numerical columns, apply optional transformations, and delete or restore samples. (D) Step 4 argument-entry interface showing method-specific parameter controls and inline help pop-ups for analysis settings, in this example “Numbers of Variables to Split” for Random Forest.

**Figure S3. Extended CytokineProfile sPLS-DA outputs for the PreT2D 20h versus 72h comparison.** (A) CytokineProfile sPLS-DA results panel showing the in-app results environment. (B) VIP score ranking for component 1. (C) ROC curve for the selected two-component model. (D) CytokineProfile’s Leave-one-out cross-validation error summary used to guide component selection. (E) MetaboAnalyst’s sPLS-DA leave-one-out cross-validation error summary used to guide component selection.

**Figure S4. Extended Random Forest and XGBoost output summaries for the PreT2D 20h versus 72h comparison.** (A) Random Forest results summary showing the held-out train/test split, class balance, hyperparameters, and performance metrics. (B) Random Forest ROC curve on the held-out test set. (C) XGBoost results summary showing the held-out train/test split, hyperparameters, feature ranking, and cross-validation summary. (D) XGBoost ROC curve on the held-out test set.
