# Supplementary material for: CytokineProfile: An Integrated Web Tool for Cytokine Profiling Analysis
Source: Comput Struct Biotechnol J. 2026 May 7;35(1):0079. doi: 10.34133/csbj.0079 (PMC13150067; doi:10.34133/csbj.0079)
Supplement: Supplementary 1 — Case Study Dataset Figs. S1 to S4 Table ST1 Notes S1 and S2 [file csbj.0079.f1.zip › SupplementaryFigures.final.pdf]

Package	Version	Package	Version	Package	Version
shiny	1.13.0	caret	7.0-1	data.table	1.18.2.1
shinyjs	2.1.1	xgboost	3.2.1.1	lifecycle	1.0.5
readxl	1.4.5	randomForest	4.7-1.2	car	3.1-5
bslib	0.10.0	pheatmap	1.0.13	Ckmeans.1d.dp	4.3.5
shinyhelper	0.3.2	e1071	1.7-17	emmeans	2.0.2
DT	0.34.0	ggplot2	4.0.2	shinytest2	0.5.1
shinyFeedback	0.4.0	ggrepel	0.9.8	spelling	2.3.2
shinyWidgets	0.9.1	ggcorrplot	0.1.4.1	BiocManager	1.30.27
shinycssloaders	1.1.0	gridExtra	2.3	testthat	3.3.2
fontawesome	0.5.3	reshape2	1.4.5	knitr	1.51
skimr	2.2.2	recipes	1.3.1	rmarkdown	2.3
mixOmics	6.32.0	impute	1.82.0	devtools	2.5.0
dplyr	1.2.0	patchwork	1.3.2	plotly	4.12.0
tidyr	1.3.2	rlang	1.1.7	learnr	0.11.6
pROC	1.19.0.1	vroom	1.7.0	htmltools	0.5.9
plot3D	1.4.2	config	0.3.2		

Table ST1

A

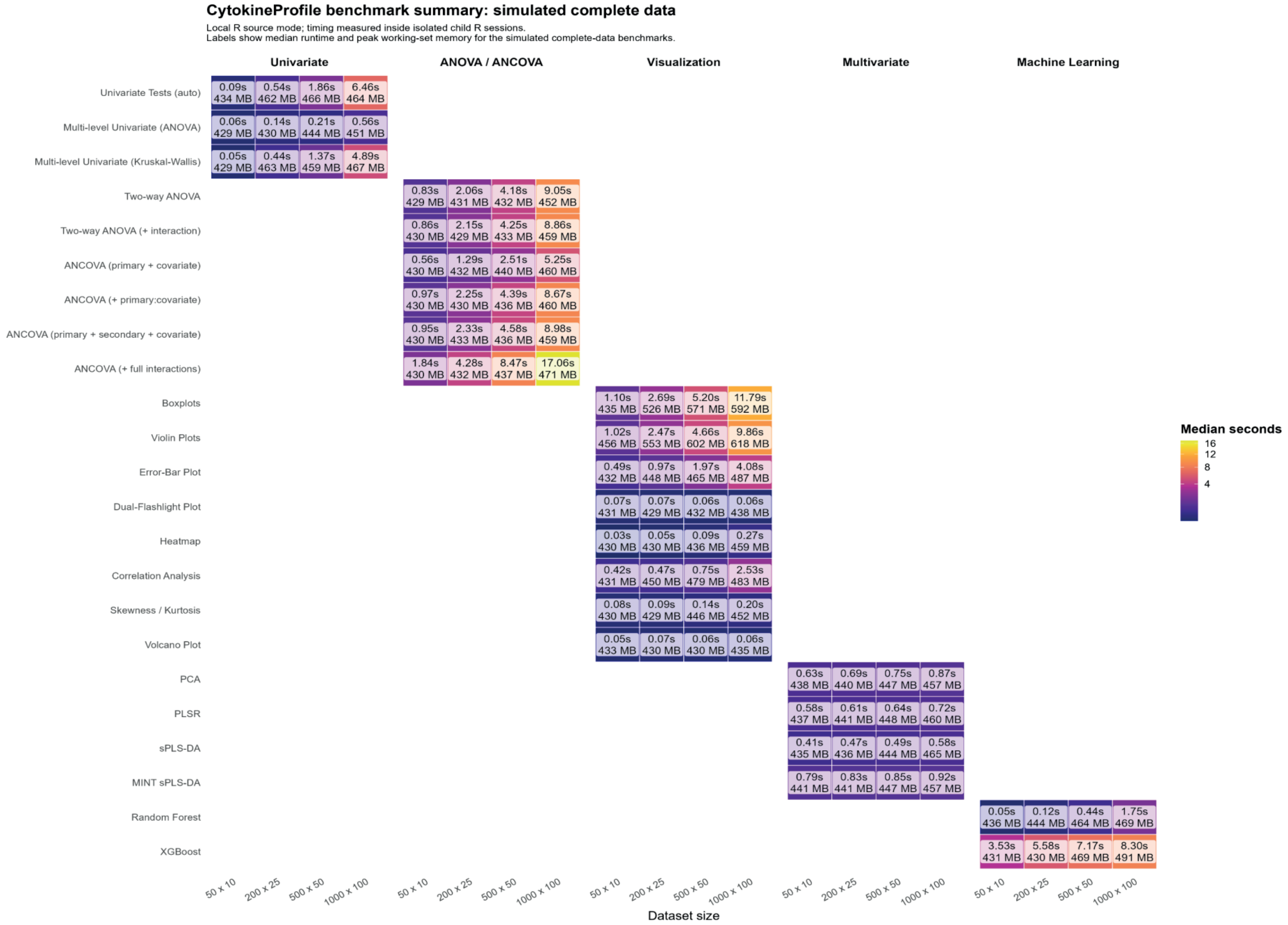
CytokineProfile benchmark summary: simulated complete data
Local R source mode; timing measured inside isolated child R sessions.
Labels show median runtime and peak working-set memory for the simulated complete-data benchmarks.
Univariate
ANOVA / ANCOVA
Visualization
Multivariate
Machine Learning
Univariate Tests (auto) 0.09s 434 MB 0.54s 462 MB 1.86s 466 MB 6.46s 464 MB
Multi-level Univariate (ANOVA) 0.06s 429 MB 0.14s 430 MB 0.21s 444 MB 0.56s 451 MB
Multi-level Univariate (Kruskal-Wallis) 0.05s 429 MB 0.44s 463 MB 1.37s 459 MB 4.89s 467 MB
Two-way ANOVA 0.83s 429 MB 2.06s 431 MB 4.18s 432 MB 9.05s 452 MB
Two-way ANOVA (+ interaction) 0.86s 430 MB 2.15s 429 MB 4.25s 433 MB 8.86s 459 MB
ANCOVA (primary + covariate) 0.56s 430 MB 1.29s 432 MB 2.51s 440 MB 5.25s 460 MB
ANCOVA (+ primary:covariate) 0.97s 430 MB 2.25s 430 MB 4.39s 436 MB 8.67s 460 MB
ANCOVA (primary + secondary + covariate) 0.95s 430 MB 2.33s 433 MB 4.58s 436 MB 8.98s 459 MB
ANCOVA (+ full interactions) 1.84s 430 MB 4.28s 432 MB 8.47s 437 MB 17.06s 471 MB
Boxplots 1.10s 435 MB 2.69s 526 MB 5.20s 571 MB 11.79s 592 MB
Violin Plots 1.02s 456 MB 2.47s 553 MB 4.66s 602 MB 9.86s 618 MB
Error-Bar Plot 0.49s 432 MB 0.97s 448 MB 1.97s 465 MB 4.08s 487 MB
Dual-Flashlight Plot 0.07s 431 MB 0.07s 429 MB 0.06s 432 MB 0.06s 438 MB
Heatmap 0.03s 430 MB 0.05s 430 MB 0.09s 436 MB 0.27s 459 MB
Correlation Analysis 0.42s 431 MB 0.47s 450 MB 0.75s 479 MB 2.53s 483 MB
Skewness / Kurtosis 0.08s 430 MB 0.09s 429 MB 0.14s 446 MB 0.20s 452 MB
Volcano Plot 0.05s 433 MB 0.07s 430 MB 0.06s 430 MB 0.06s 435 MB
PCA 0.63s 438 MB 0.69s 440 MB 0.75s 447 MB 0.87s 457 MB
PLSR 0.58s 437 MB 0.61s 441 MB 0.64s 448 MB 0.72s 460 MB
sPLS-DA 0.41s 435 MB 0.47s 436 MB 0.49s 444 MB 0.58s 465 MB
MINT sPLS-DA 0.79s 441 MB 0.83s 441 MB 0.85s 447 MB 0.92s 457 MB
Random Forest 0.05s 436 MB 0.12s 444 MB 0.44s 464 MB 1.75s 469 MB
XGBoost 3.53s 431 MB 5.58s 430 MB 7.17s 469 MB 8.30s 491 MB
50 x 10
200 x 25
500 x 50
1000 x 100
Dataset size
Median seconds
16
12
8
4

Figure S1

B

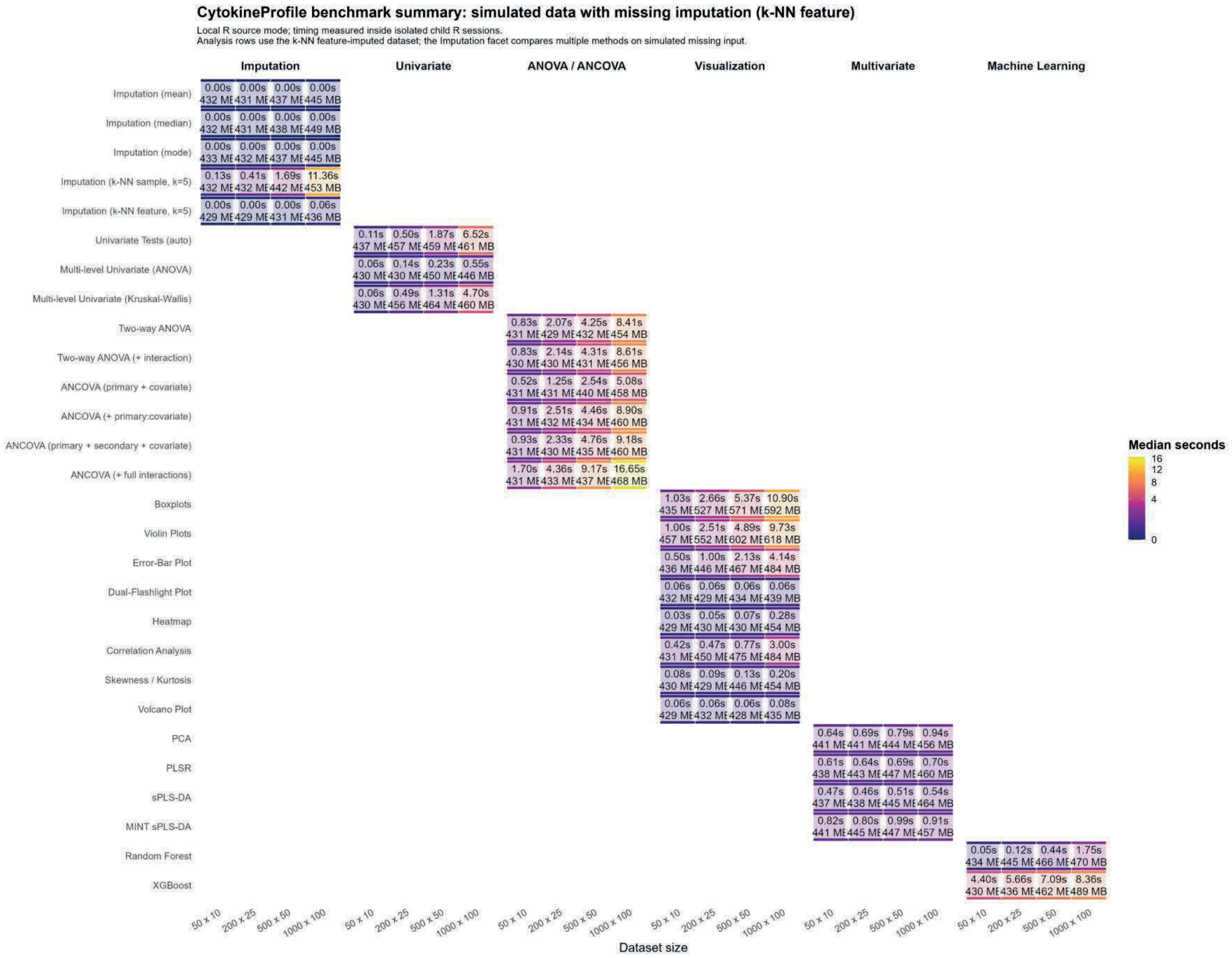
CytokineProfile benchmark summary: simulated data with missing imputation (k-NN feature)
Local R source mode; timing measured inside isolated child R sessions.
Analysis rows use the k-NN feature-imputed dataset; the Imputation facet compares multiple methods on simulated missing input.
Imputation
Univariate
ANOVA / ANCOVA
Visualization
Multivariate
Machine Learning
Imputation (mean) 0.00s 432 ME 0.00s 431 ME 0.00s 437 ME 0.00s 445 MB
Imputation (median) 0.00s 432 ME 0.00s 431 ME 0.00s 438 ME 0.00s 449 MB
Imputation (mode) 0.00s 433 ME 0.00s 432 ME 0.00s 437 ME 0.00s 445 MB
Imputation (k-NN sample, k=5) 0.13s 432 ME 0.41s 432 ME 1.69s 442 ME 11.36s 453 MB
Imputation (k-NN feature, k=5) 0.00s 429 ME 0.00s 429 ME 0.00s 431 ME 0.06s 436 MB
Univariate Tests (auto) 0.11s 437 ME 0.50s 457 ME 1.87s 459 ME 6.52s 461 MB
Multi-level Univariate (ANOVA) 0.06s 430 ME 0.14s 430 ME 0.23s 450 ME 0.55s 446 MB
Multi-level Univariate (Kruskal-Wallis) 0.06s 430 ME 0.49s 456 ME 1.31s 464 ME 4.70s 460 MB
Two-way ANOVA 0.83s 431 ME 2.07s 429 ME 4.25s 432 ME 8.41s 454 MB
Two-way ANOVA (+ interaction) 0.83s 430 ME 2.14s 430 ME 4.31s 431 ME 8.61s 456 MB
ANCOVA (primary + covariate) 0.52s 431 ME 1.25s 431 ME 2.54s 440 ME 5.08s 458 MB
ANCOVA (+ primary:covariate) 0.91s 431 ME 2.51s 432 ME 4.46s 434 ME 8.90s 460 MB
ANCOVA (primary + secondary + covariate) 0.93s 431 ME 2.33s 430 ME 4.76s 435 ME 9.18s 460 MB
ANCOVA (+ full interactions) 1.70s 431 ME 4.36s 433 ME 9.17s 437 ME 16.65s 468 MB
Boxplots 1.03s 435 ME 2.66s 527 ME 5.37s 571 ME 10.90s 592 MB
Violin Plots 1.00s 457 ME 2.51s 552 ME 4.89s 602 ME 9.73s 618 MB
Error-Bar Plot 0.50s 436 ME 1.00s 446 ME 2.13s 467 ME 4.14s 484 MB
Dual-Flashlight Plot 0.06s 432 ME 0.06s 429 ME 0.06s 434 ME 0.06s 439 MB
Heatmap 0.03s 429 ME 0.05s 430 ME 0.07s 430 ME 0.28s 454 MB
Correlation Analysis 0.42s 431 ME 0.47s 450 ME 0.77s 475 ME 3.00s 484 MB
Skewness / Kurtosis 0.08s 430 ME 0.09s 429 ME 0.13s 446 ME 0.20s 454 MB
Volcano Plot 0.06s 429 ME 0.06s 432 ME 0.06s 428 ME 0.08s 435 MB
PCA 0.64s 441 ME 0.69s 441 ME 0.79s 444 ME 0.94s 456 MB
PLSR 0.61s 438 ME 0.64s 443 ME 0.69s 447 ME 0.70s 460 MB
sPLS-DA 0.47s 437 ME 0.46s 438 ME 0.51s 445 ME 0.54s 464 MB
MINT sPLS-DA 0.82s 441 ME 0.80s 445 ME 0.99s 447 ME 0.91s 457 MB
Random Forest 0.05s 434 ME 0.12s 445 ME 0.44s 466 ME 1.75s 470 MB
XGBoost 4.40s 430 ME 5.66s 436 ME 7.09s 462 ME 8.36s 489 MB
50 x 10
200 x 25
500 x 50
1000 x 100
Dataset size
Median seconds
16
12
8
4
0

Figure S1

A

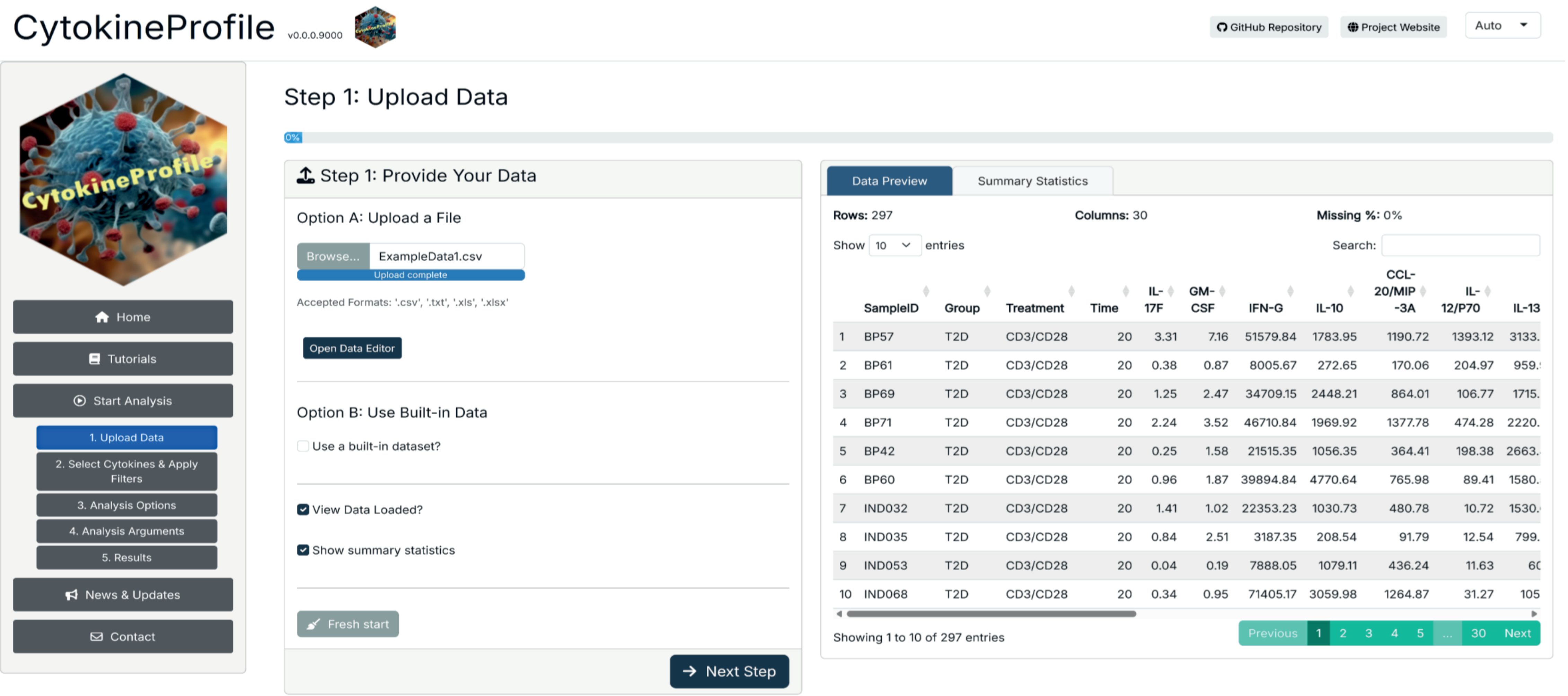
CytokineProfile
v0.0.0.9000
GitHub Repository
Project Website
Auto
Home
Tutorials
Start Analysis
1. Upload Data
2. Select Cytokines & Apply Filters
3. Analysis Options
4. Analysis Arguments
5. Results
News & Updates
Contact
Step 1: Upload Data
0%
Step 1: Provide Your Data
Option A: Upload a File
Browse...
ExampleData1.csv
Upload complete
Accepted Formats: '.csv', '.txt', '.xls', '.xlsx'
Open Data Editor
Option B: Use Built-in Data
Use a built-in dataset?
View Data Loaded?
Show summary statistics
Fresh start
Next Step
Data Preview
Summary Statistics
Rows: 297
Columns: 30
Missing %: 0%
Show 10 entries
Search:
SampleID Group Treatment Time IL-17F GM-CSF IFN-G IL-10 CCL-20/MIP-3A IL-12/P70 IL-13
1 BP57 T2D CD3/CD28 20 3.31 7.16 51579.84 1783.95 1190.72 1393.12 3133.
2 BP61 T2D CD3/CD28 20 0.38 0.87 8005.67 272.65 170.06 204.97 959.
3 BP69 T2D CD3/CD28 20 1.25 2.47 34709.15 2448.21 864.01 106.77 1715.
4 BP71 T2D CD3/CD28 20 2.24 3.52 46710.84 1969.92 1377.78 474.28 2220.
5 BP42 T2D CD3/CD28 20 0.25 1.58 21515.35 1056.35 364.41 198.38 2663.
6 BP60 T2D CD3/CD28 20 0.96 1.87 39894.84 4770.64 765.98 89.41 1580.
7 IND032 T2D CD3/CD28 20 1.41 1.02 22353.23 1030.73 480.78 10.72 1530.
8 IND035 T2D CD3/CD28 20 0.84 2.51 3187.35 208.54 91.79 12.54 799.
9 IND053 T2D CD3/CD28 20 0.04 0.19 7888.05 1079.11 436.24 11.63 60
10 IND068 T2D CD3/CD28 20 0.34 0.95 71405.17 3059.98 1264.87 31.27 105
Showing 1 to 10 of 297 entries
Previous 1 2 3 4 5 ... 30 Next

Figure S2

B

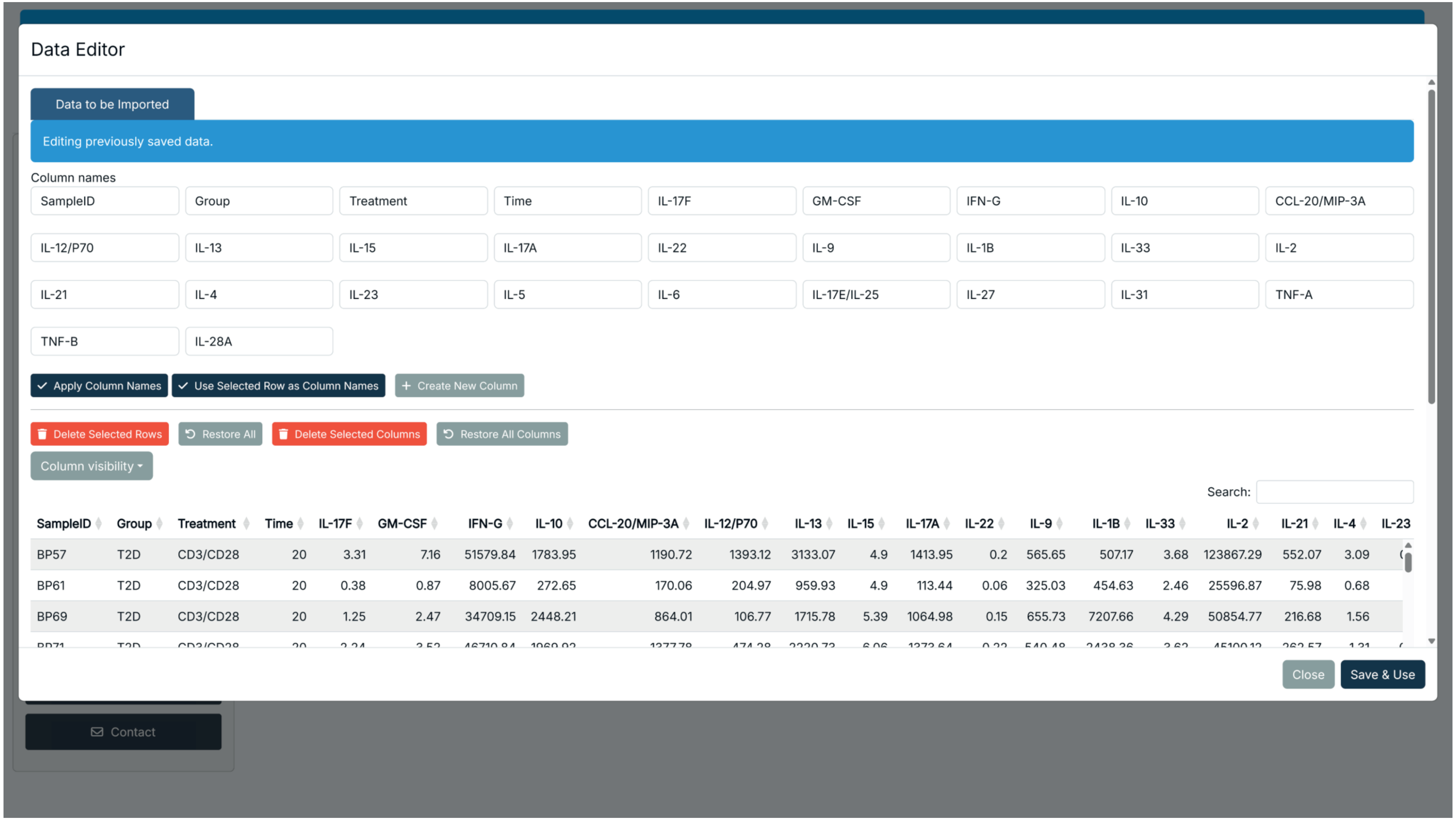

Data Editor
Data to be Imported
Editing previously saved data.
Column names
SampleID
Group
Treatment
Time
IL-17F
GM-CSF
IFN-G
IL-10
CCL-20/MIP-3A
IL-12/P70
IL-13
IL-15
IL-17A
IL-22
IL-9
IL-1B
IL-33
IL-2
IL-21
IL-4
IL-23
IL-5
IL-6
IL-17E/IL-25
IL-27
IL-31
TNF-A
TNF-B
IL-28A
Apply Column Names
Use Selected Row as Column Names
Create New Column
Delete Selected Rows
Restore All
Delete Selected Columns
Restore All Columns
Column visibility
Search:
SampleID Group Treatment Time IL-17F GM-CSF IFN-G IL-10 CCL-20/MIP-3A IL-12/P70 IL-13 IL-15 IL-17A IL-22 IL-9 IL-1B IL-33 IL-2 IL-21 IL-4 IL-23
BP57 T2D CD3/CD28 20 3.31 7.16 51579.84 1783.95 1190.72 1393.12 3133.07 4.9 1413.95 0.2 565.65 507.17 3.68 123867.29 552.07 3.09
BP61 T2D CD3/CD28 20 0.38 0.87 8005.67 272.65 170.06 204.97 959.93 4.9 113.44 0.06 325.03 454.63 2.46 25596.87 75.98 0.68
BP69 T2D CD3/CD28 20 1.25 2.47 34709.15 2448.21 864.01 106.77 1715.78 5.39 1064.98 0.15 655.73 7207.66 4.29 50854.77 216.68 1.56
Close
Save & Use
Contact

Figure S2

C

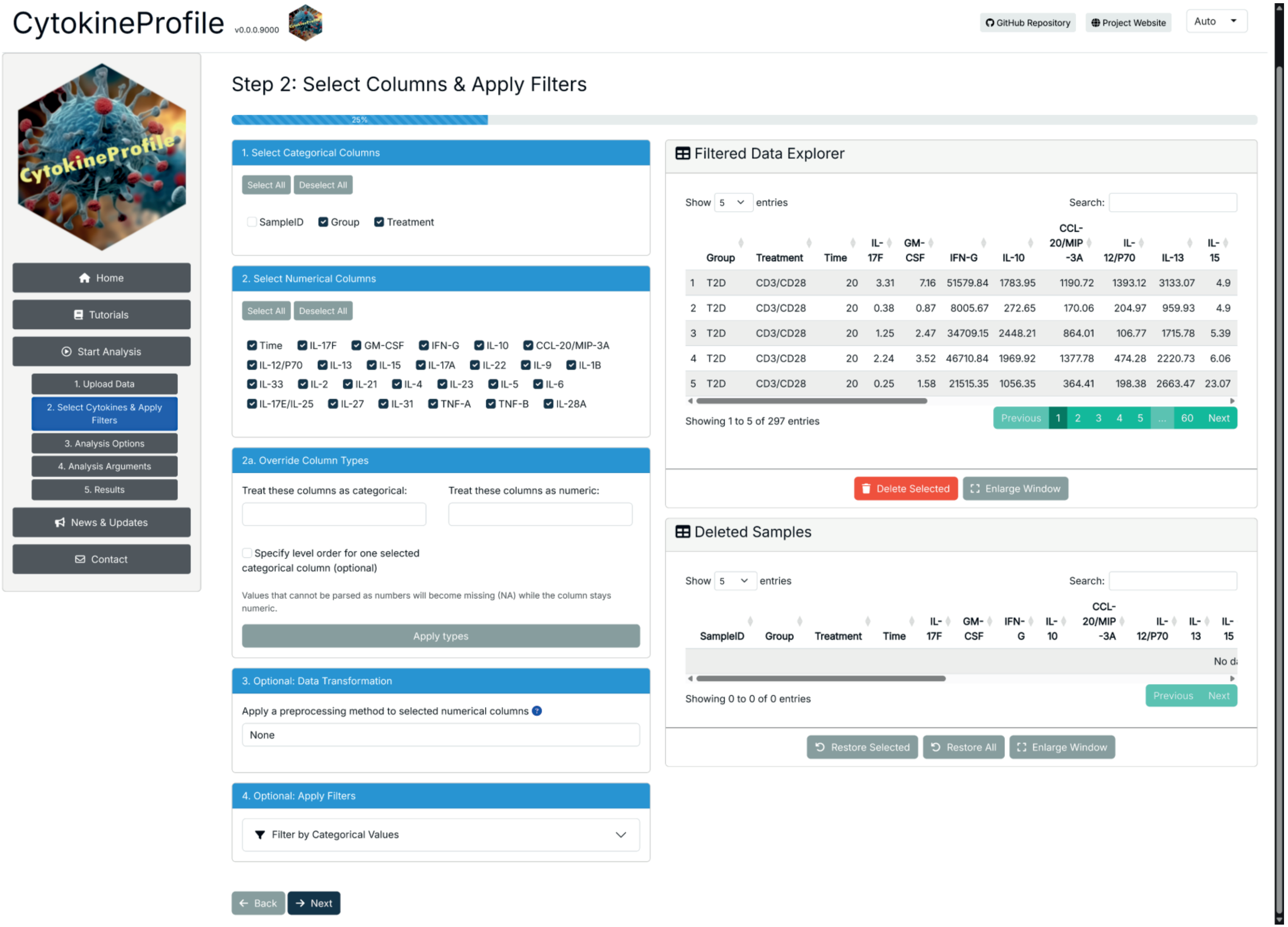
CytokineProfile v0.0.0.9000
GitHub Repository
Project Website
Auto
Step 2: Select Columns & Apply Filters
25%
Home
Tutorials
Start Analysis
1. Upload Data
2. Select Cytokines & Apply Filters
3. Analysis Options
4. Analysis Arguments
5. Results
News & Updates
Contact
1. Select Categorical Columns
Select All
Deselect All
SampleID
Group
Treatment
2. Select Numerical Columns
Time
IL-17F
GM-CSF
IFN-G
IL-10
CCL-20/MIP-3A
IL-12/P70
IL-13
IL-15
IL-17A
IL-22
IL-9
IL-1B
IL-33
IL-2
IL-21
IL-4
IL-23
IL-5
IL-6
IL-17E/IL-25
IL-27
IL-31
TNF-A
TNF-B
IL-28A
2a. Override Column Types
Treat these columns as categorical:
Treat these columns as numeric:
Specify level order for one selected categorical column (optional)
Values that cannot be parsed as numbers will become missing (NA) while the column stays numeric.
Apply types
3. Optional: Data Transformation
Apply a preprocessing method to selected numerical columns
None
4. Optional: Apply Filters
Filter by Categorical Values
Back
Next
Filtered Data Explorer
Show 5 entries
Search:
Group Treatment Time IL-17F GM-CSF IFN-G IL-10 CCL-20/MIP-3A IL-12/P70 IL-13 IL-15
1 T2D CD3/CD28 20 3.31 7.16 51579.84 1783.95 1190.72 1393.12 3133.07 4.9
2 T2D CD3/CD28 20 0.38 0.87 8005.67 272.65 170.06 204.97 959.93 4.9
3 T2D CD3/CD28 20 1.25 2.47 34709.15 2448.21 864.01 106.77 1715.78 5.39
4 T2D CD3/CD28 20 2.24 3.52 46710.84 1969.92 1377.78 474.28 2220.73 6.06
5 T2D CD3/CD28 20 0.25 1.58 21515.35 1056.35 364.41 198.38 2663.47 23.07
Showing 1 to 5 of 297 entries
Previous 1 2 3 4 5 … 60 Next
Delete Selected
Enlarge Window
Deleted Samples
Show 5 entries
Search:
SampleID Group Treatment Time IL-17F GM-CSF IFN-G IL-10 CCL-20/MIP-3A IL-12/P70 IL-13 IL-15
Showing 0 to 0 of 0 entries
Previous Next
Restore Selected
Restore All
Enlarge Window

Figure S2

D

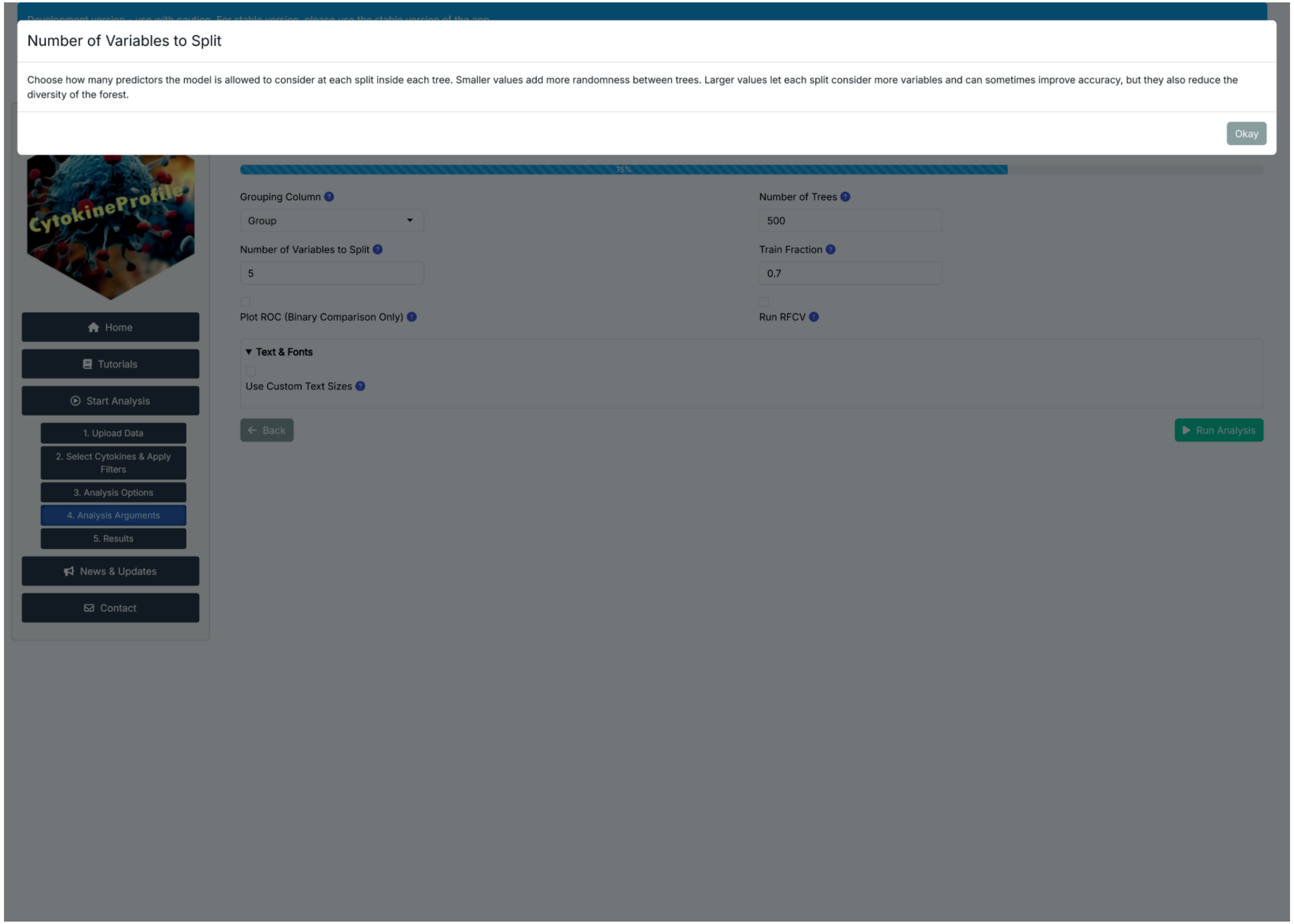
Number of Variables to Split
Choose how many predictors the model is allowed to consider at each split inside each tree. Smaller values add more randomness between trees. Larger values let each split consider more variables and can sometimes improve accuracy, but they also reduce the diversity of the forest.
Okay
CytokineProfile
Home
Tutorials
Start Analysis
1. Upload Data
2. Select Cytokines & Apply Filters
3. Analysis Options
4. Analysis Arguments
5. Results
News & Updates
Contact
Grouping Column
Group
Number of Trees
500
Number of Variables to Split
5
Train Fraction
0.7
Plot ROC (Binary Comparison Only)
Run RFCV
Text & Fonts
Use Custom Text Sizes
Back
Run Analysis

Figure S2

A

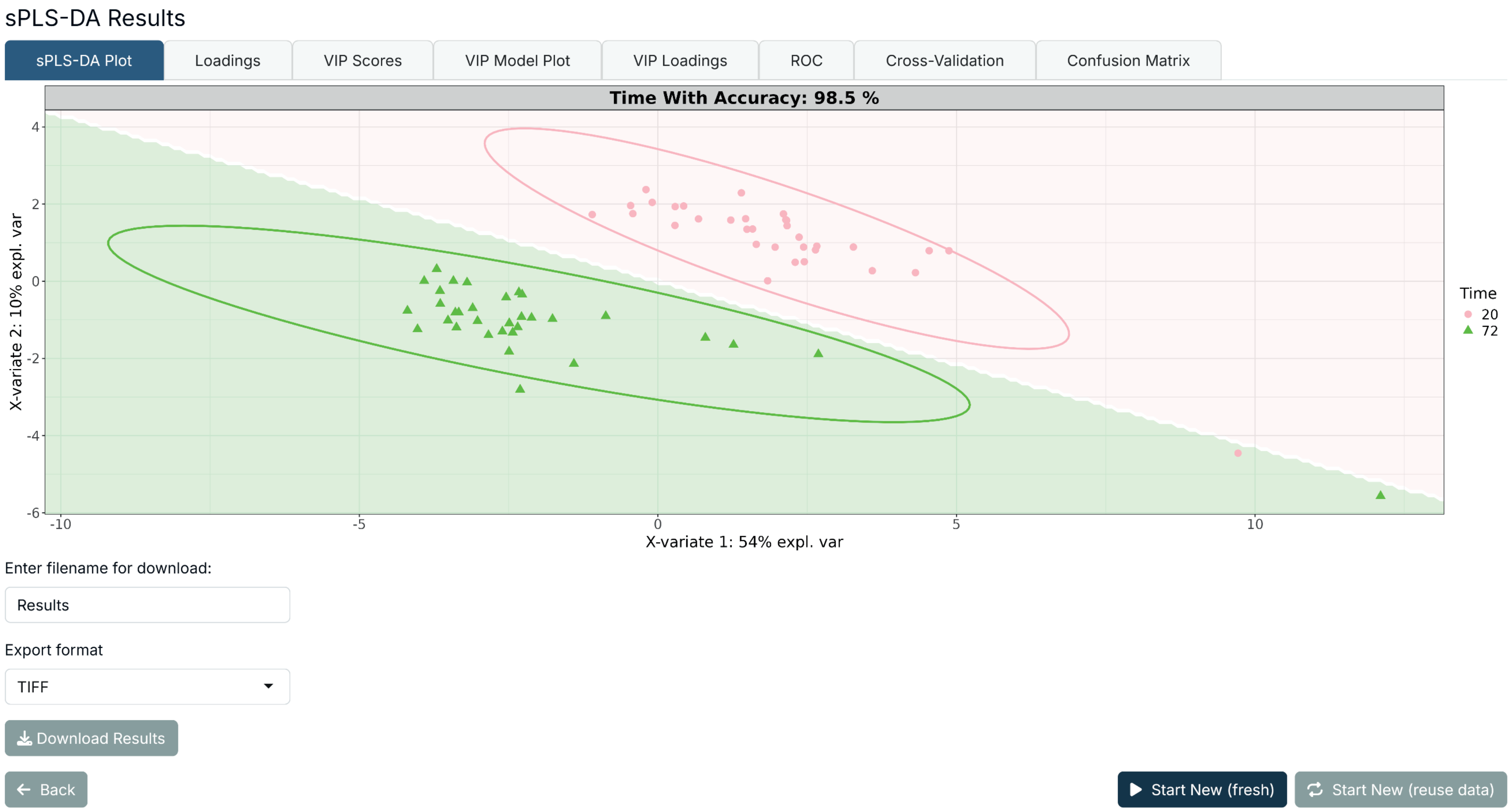
sPLS-DA Results
sPLS-DA Plot
Loadings
VIP Scores
VIP Model Plot
VIP Loadings
ROC
Cross-Validation
Confusion Matrix
Time With Accuracy: 98.5 %
X-variate 2: 10% expl. var
X-variate 1: 54% expl. var
4
2
0
-2
-4
-6
-10
-5
0
5
10
Time
20
72
Enter filename for download:
Results
Export format
TIFF
Download Results
Back
Start New (fresh)
Start New (reuse data)

Figure S3

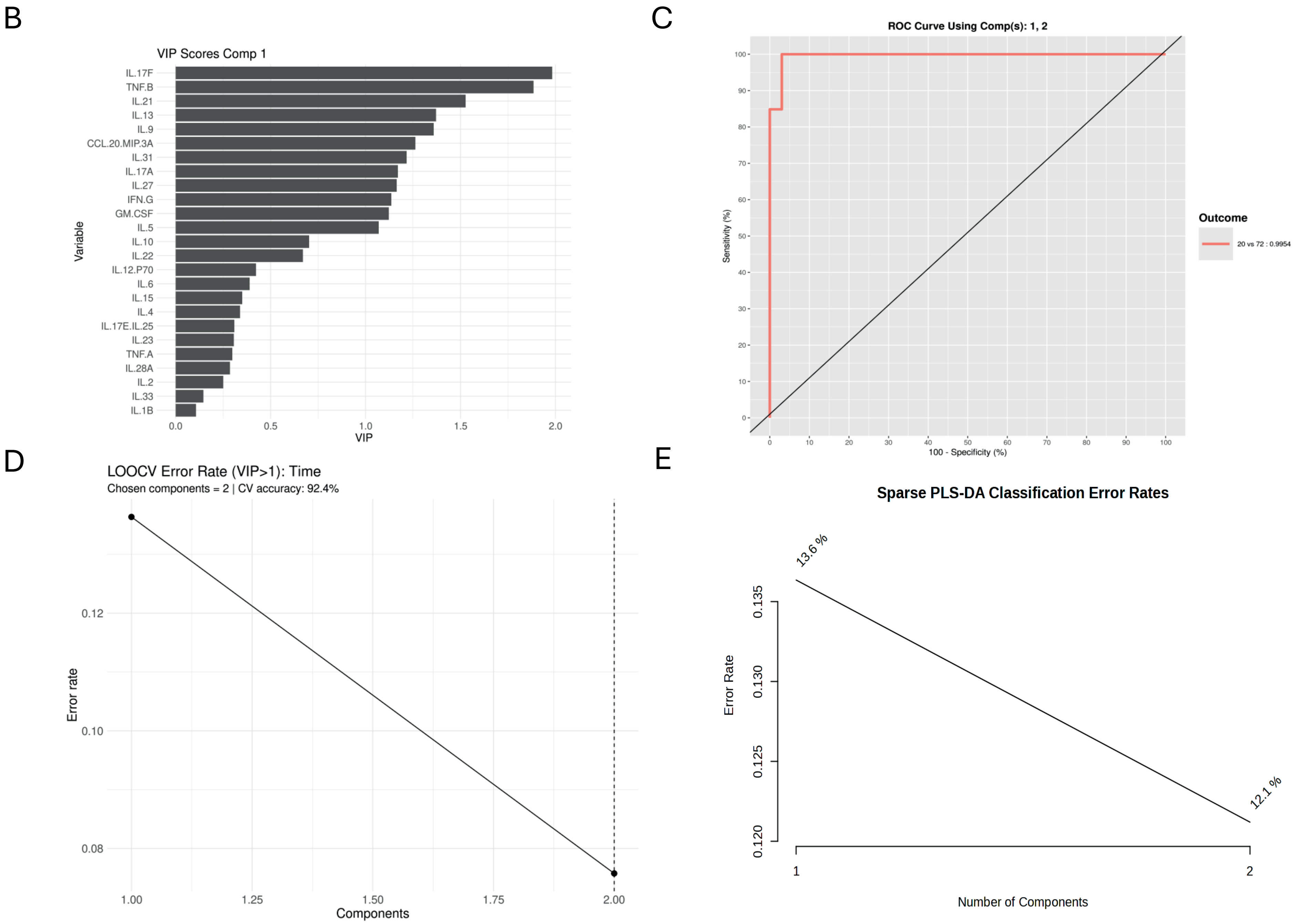
B
VIP Scores Comp 1
Variable
IL.17F
TNF.B
IL.21
IL.13
IL.9
CCL.20.MIP.3A
IL.31
IL.17A
IL.27
IFN.G
GM.CSF
IL.5
IL.10
IL.22
IL.12.P70
IL.6
IL.15
IL.4
IL.17E.IL.25
IL.23
TNF.A
IL.28A
IL.2
IL.33
IL.1B
0.0
0.5
1.0
1.5
2.0
VIP
C
ROC Curve Using Comp(s): 1, 2
Sensitivity (%)
100 - Specificity (%)
Outcome
20 vs 72 : 0.9954
D
LOOCV Error Rate (VIP>1): Time
Chosen components = 2 | CV accuracy: 92.4%
Error rate
0.12
0.10
0.08
1.00
1.25
1.50
1.75
2.00
Components
E
Sparse PLS-DA Classification Error Rates
Error Rate
0.135
0.130
0.125
0.120
13.6 %
12.1 %
1
2
Number of Components

Figure S3

A

Random Forest Results

Summary | Variable Importance | ROC Curve | Cross-Validation

Copy

```
### RANDOM FOREST RESULTS ###

--- Analysis Design ---
Performance provenance: holdout train/test split.
Samples after filtering: 66
Predictors used: 12
Requested train fraction: 0.7
Observed split: train = 48 , test = 18
Class balance (all data): 20=33 (50%), 72=33 (50%)
Class balance (train): 20=24 (50%), 72=24 (50%)
Class balance (test): 20=9 (50%), 72=9 (50%)
Seed: 123456
Hyperparameters: ntree = 500 , mtry = 5
RFCV enabled: Yes
RFCV folds: 5
RFCV step: 0.5
Caret CV enabled: No

--- Training Set ---
Confusion Matrix:
          Reference
Prediction 20 72
        20 24  0
        72  0 24

Accuracy: 1

Sensitivity (train): 1
Specificity (train): 1

--- Test Set ---
Confusion Matrix:
          Reference
Prediction 20 72
        20  8  1
        72  1  8

Accuracy: 0.889

AUC: 0.975

Sensitivity (test): 0.889
Specificity (test): 0.889
```

Figure S4

B

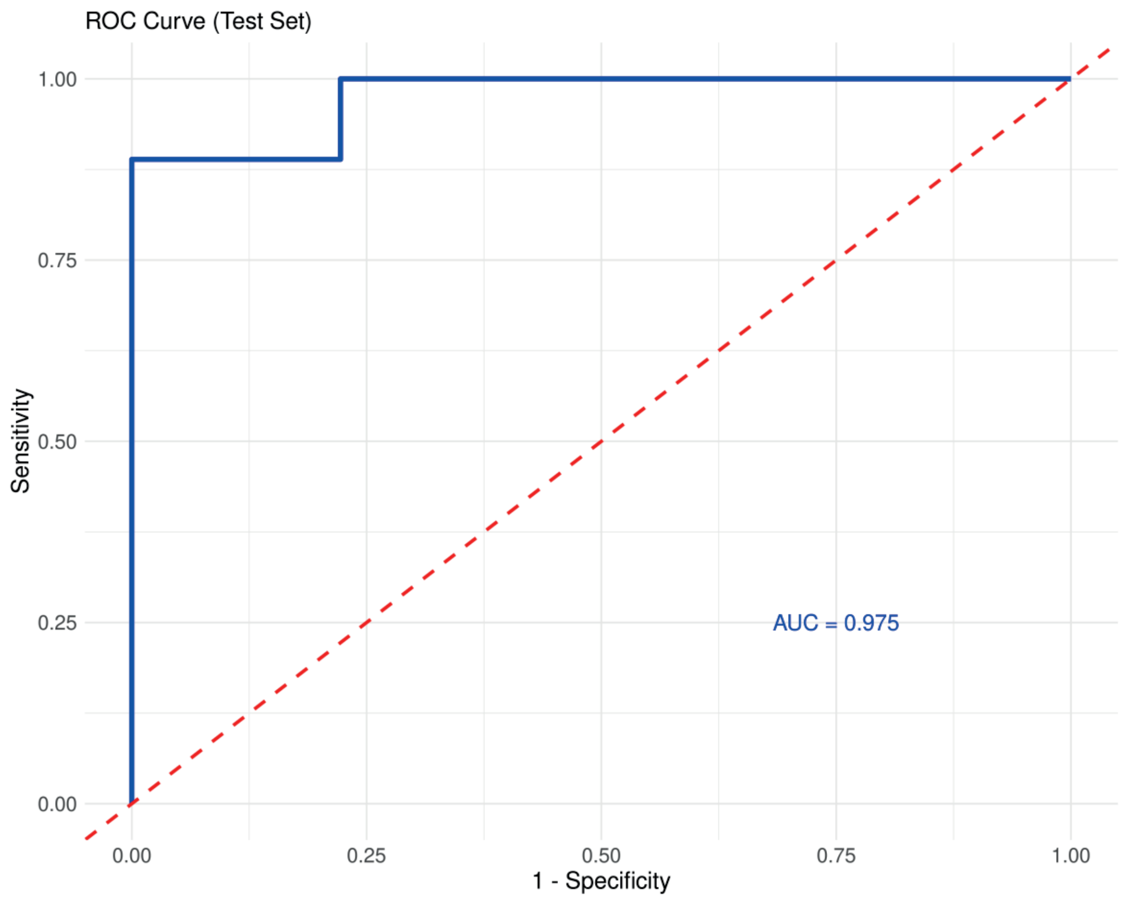

ROC Curve (Test Set)
1.00
0.75
0.50
0.25
0.00
Sensitivity
0.00
0.25
0.50
0.75
1.00
1 - Specificity
AUC = 0.975

Figure S4

C

XGBoost Results

Summary | Variable Importance | ROC Curve

Copy

```
### XGBOOST RESULTS ###

1) Analysis Design:
Performance provenance: holdout train/test split.
Samples after filtering: 66
Predictors used: 12
Requested train fraction: 0.7
Observed split: train = 48 , test = 18
Class balance (all data): 20=33 (50%), 72=33 (50%)
Class balance (train): 20=24 (50%), 72=24 (50%)
Class balance (test): 20=9 (50%), 72=9 (50%)
Seed: 123456
Hyperparameters: nrounds = 500 , max_depth = 4 , learning_rate = 0.1 , min_split_loss = 0 , colsample_bytree = 1 , subsample = 1 , min_child_weight = 1
Objective: multi:softprob
Evaluation metric: auc
Early stopping rounds: None
Cross-validation enabled: Yes
Cross-validation folds: 5

2) Group -> Numeric Label Mapping:
20 72
 0  1

3) Confusion Matrix on Test Set:
          Reference
Prediction 0 1
         0 8 1
         1 1 8

Test Accuracy: 0.889

Sensitivity: 0.889
Specificity: 0.889

AUC: 0.951

4) Top 12 Important Features:
          Feature        Gain       Cover   Frequency
           <char>       <num>       <num>       <num>
 1:        IL.17F 0.933779751 0.367355118 0.180811808
 2: CCL.20.MIP.3A 0.021258094 0.135997959 0.164206642
 3:         IL.21 0.012415850 0.027360160 0.011070111
 4:         IFN.G 0.006625307 0.069874909 0.090405904
 5:         IL.13 0.005296416 0.010430631 0.011070111
 6:        GM.CSF 0.004957519 0.042069673 0.049815498
 7:         TNF.B 0.004871152 0.122785684 0.175276753
 8:          IL.9 0.003785276 0.025644401 0.033210332
 9:         IL.31 0.002451362 0.164452565 0.239852399
10:         IL.27 0.002328846 0.003730622 0.003690037
11:        IL.17A 0.001412820 0.017358410 0.023985240
12:          IL.5 0.000817608 0.012939869 0.016605166

5) Cross-Validation Results:
CV provenance: xgb.cv run on the training split only.
CV Accuracy: NA
Best iteration:
    iter train_auc_mean train_auc_std test_auc_mean test_auc_std
   <int>          <num>         <num>         <num>        <num>
1:     1      0.9981101   0.001199698     0.9714286   0.06388766
```

Figure S4

D

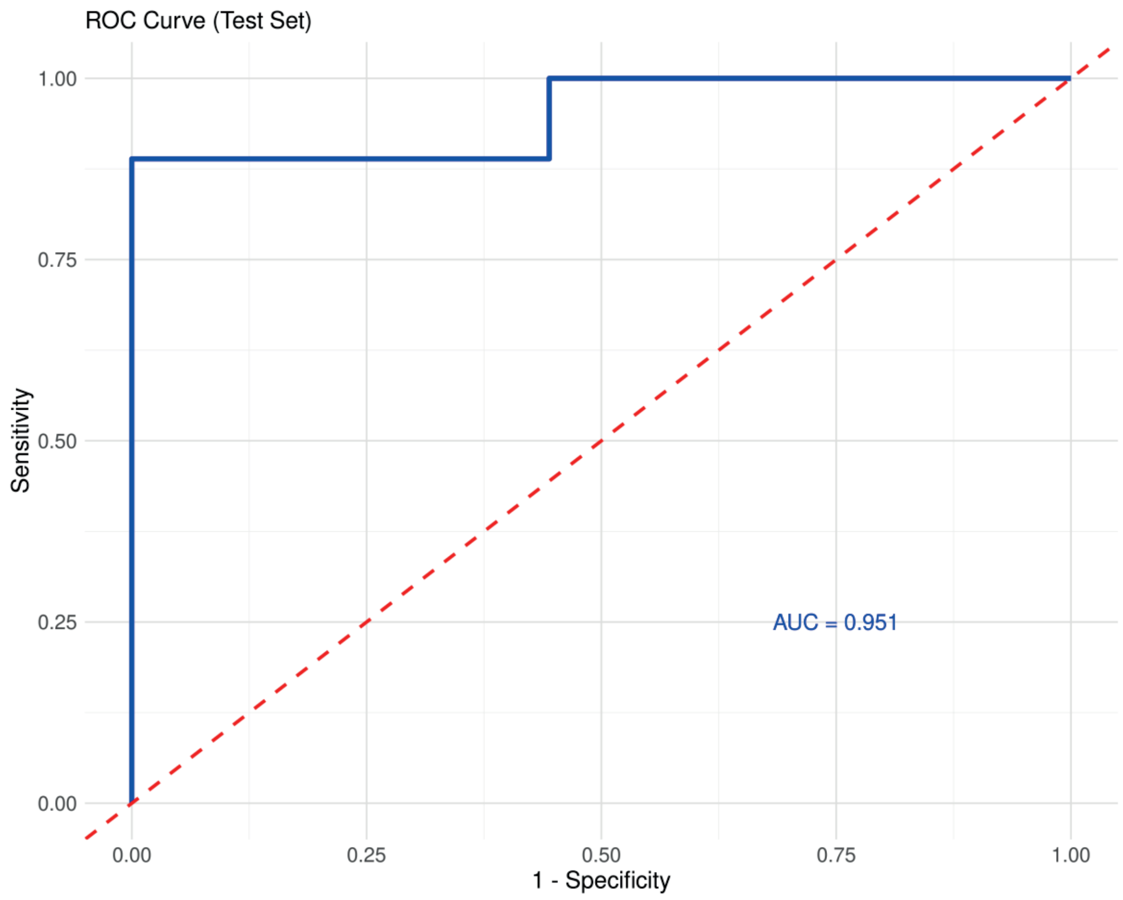
ROC Curve (Test Set)
Sensitivity
1.00
0.75
0.50
0.25
0.00
0.00
0.25
0.50
0.75
1.00
1 - Specificity
AUC = 0.951

Figure S4
